# Membrane-association of EMR2/ADGRE2-NTF is regulated by site-specific N-glycosylation

**DOI:** 10.1038/s41598-018-22849-x

**Published:** 2018-03-14

**Authors:** Yi-Shu Huang, Nien-Yi Chiang, Gin-Wen Chang, Hsi-Hsien Lin

**Affiliations:** 1 Department of Microbiology and Immunology, College of Medicine, Chang Gung University, Taoyuan, Taiwan; 2 Department of Anatomic Pathology, Chang Gung Memorial Hospital-Linkou, Taoyuan, Taiwan; 3 Chang Gung Immunology Consortium, Chang Gung Memorial Hospital-Linkou, Taoyuan, Taiwan; 4 Present Address: Kennedy Institute of Rheumatology, University of Oxford, OX3 7FY Oxford, UK

## Abstract

The evolutionarily conserved adhesion G protein-coupled receptors (aGPCRs) play critical roles in biological processes as diverse as brain development, cell polarity and innate immune functions. A defining feature of aGPCRs is the GPCR autoproteolysis inducing (GAIN) domain capable of self-catalytic cleavage, resulting in the generation of an extracellular N-terminal fragment (NTF) and a seven-transmembrane C-terminal fragment (CTF) involved in the cellular adhesion and signaling functions, respectively. Interestingly, two different NTF subtypes have previously been identified, namely an NTF that couples non-covalently with the CTF and a membrane-associated NTF that tethers on cell surface independently. The two NTF subtypes are expected to regulate aGPCR signaling via distinct mechanisms however their molecular characteristics are largely unknown. Herein, the membrane-associated NTF of EMR2/ADGRE2 is investigated and found to be modified by differential N-glycosylation. The membrane association of EMR2-NTF occurs in post-ER compartments and site-specific N-glycosylation in the GAIN domain is involved in modulating its membrane-association ability. Finally, a unique amphipathic α-helix in the GAIN domain is identified as a putative membrane anchor of EMR2-NTF. These results provide novel insights into the complex interaction and activation mechanisms of aGPCRs.

## Introduction

Characterized by a long extracellular domain (ECD) with cell-adhesion functions and a seven-transmembrane (7TM) domain with signaling functions, the adhesion G protein-coupled receptors (aGPCRs) have been implicated in diverse biological activities and human diseases^1^. These include control of cellular polarity^2,3^, development of human frontal cortex, oligodendrocyte and Schwann cell^4–7^, CNS angiogenesis and blood-brain-barrier integrity^8–10^, epididymal fluid regulation and male fertility^11,12^, as well as innate immune regulation^13–15^.

The hallmark of aGPCRs is the presence of a conserved GPCR proteolysis site (GPS) motif located at the C-terminal end of a much larger GPCR autoproteolysis inducing (GAIN) domain^1^. Molecular and functional studies have highlighted the importance of this unusual post-translational auto-proteolytic processing and the resulting dual-subunit receptor organization in aGPCR-mediated functions^16^. Nevertheless, more recent *in vivo* studies have shown that the GPS auto-proteolysis might not be essential for the physiological functions of certain aGPCRs^17,18^. Structural analysis indicates that the GAIN domain is an evolutionally-conserved auto-proteolytic protein fold, which is subdivided into a sub-domain A with six α-helices and a sub-domain B with 13 β-strands and 2 small α-helices^19^. During its biosynthesis, the receptor is self-catalytically cleaved into a GAIN domain-containing N-terminal fragment (NTF) and a 7TM domain-containing C-terminal fragment (CTF) that starts with the 13^th^ β-strand of sub-domain B^16,20^. However, it is structurally unfavorable for the two subunits to dissociate as the β-strand of CTF forms an integral part of the GAIN domain. Hence, the NTF and CTF usually remain as a non-covalent hetero-dimeric complex on the cell surface^1^.

Diverse activation models have been proposed for aGPCRs, but increasing evidence indicate that the dissociation of the NTF-CTF complex is likely the most prevalent activation mechanism^21,22^. Indeed, it was found that following the separation of the NTF from CTF the newly exposed N-terminus of the CTF in turn acted as a tethered agonist to activate the own CTF. The tethered agonism of aGPCRs, akin to the activation of protease-activated receptors (PARs), has been confirmed in several *in vitro* structure-function studies and backed up by *in vivo* experiments and human genetic disorders such as vibratory urticaria^13,23–25^. Furthermore, the so called Stachel peptide at the N-terminus of the CTF (i.e. the 13^th^ β-strand of sub-domain B) has been used successfully as a receptor-specific agonist^26^. It is likely that as a result of the engagement of NTF with its binding partners (ligands) in combination with shear force or mechanical disturbance, the NTF is detached from the CTF leading to the exposure and binding of the tethered Stachel peptide for aGPCR activation^27^. In light of this, some aGPCRs have been considered as potential metabotropic mechanosensors^16,28,29^. Nonetheless, not all aGPCRs undergo GPS proteolysis and non-cleavable aGPCRs are still able to be activated^30^. Indeed, both Stachel-dependent and -independent activation mechanisms have been shown and GPS proteolysis was shown in a recent publication functionally unnecessary for the cleavable *Drosophila* latrophilin (dCIRL) to initiate cellular mechano-signaling^17,31–33^. These recent advances suggest that aGPCR activation mechanisms are more versatile than what was thought previously. Regardless, the current consensus is that the strength and mode of interaction between the NTF and CTF seem to play a key role in modulating the activation and signaling activities of aGPCRs^30,34^.

In this context, of interest is an alternative aGPCR activation mechanism, the split personality hypothesis, whereby the NTF and CTF are dissociated but remain as independent entities on the membrane^35^. Indeed, we and others have found in EMR2/ADGRE2 and latrophilin-1/ ADGRL1 that while the majority of the NTF forms a non-covalent complex with the CTF, minor fractions of NTFs are able to dissociate from the CTF and associate independently on the membrane^36,37^. Although significant evidences for the presence of the membrane-associated NTF have been presented, the molecular characteristics of the two NTF forms remain largely unknown. In this report, the membrane association of EMR2-NTF is investigated. Our results show that the membrane-associated EMR2-NTF displays a distinct N-glycosylation pattern. Moreover, its membrane-association ability is found to be regulated by site-specific N-glycosylation in the GAIN domain. We also show that the membrane-association of EMR2-NTF takes place in post-ER compartments and identify a unique amphipathic α-helix at the GAIN sub-domain A as a putative in-plane membrane anchor. Our findings shed lights on a divergent NTF form of aGPCRs and its functional implication in modulating aGPCR activities is discussed.

## Materials and Methods

### Reagents and antibodies

General reagents were obtained from Sigma-Aldrich (Gillingham, Dorset, UK) or BDH-Merck (Poole, Dorset, UK) unless specified otherwise. Oligonucleotide primers were supplied by Tri-I Biotech (Taipei, Taiwan). DNA and protein reagents were obtained from Invitrogen (Carlsbad, CA), Qiagen (Valencia, CA), Fermentas (ON, Canada), New England Biolabs (MA, USA), or Amersham (GE Healthcare). Antibodies (Abs) used in the study are: EMR2 stalk-specific 2A1 from AbD Serotec (Kidlington, UK); Anti-CD71 (H68.4) from Zymed Laboratories (S. Francisco, CA, USA); Anti-β-actin (clone C4) and Chicken anti-DAF (CD55) polyclonal Abs from Chemicon (California, USA); Fluorochrome-conjugated goat anti-mouse IgG was from Jackson ImmunoResearch (West Grove, PA, USA); Mouse IgG_1_ isotype control (clone 11711) was from R&D Systems (MN, USA); Anti-Myc was from Invitrogen; Horseradish peroxidase (HRP)-conjugated goat anti-mouse IgG, anti-G_αi1,2_ and anti-G_β_ were from Sigma-Aldrich; Anti-EMR2/CTF-subunit polyclonal Ab has been described previously^37^.

### Cell culture and transient transfection

All cell culture media and supplements including 10% heat inactivated fetal calf serum (FCS), 2 mM L-glutamine, 50 IU/ml penicillin and 50 μg/ml streptomycin were purchased from Invitrogen. CHO-K1 and HEK293T cells were cultured in Ham’s F-12 and Dulbecco’s modified Eagle’s medium (DMEM), respectively. Transient transfection of expression constructs was performed using Lipofectamine^TM^ (Invitrogen) reagent as described previously^38^. Cells were washed 6 hr post transfection and cultured with fresh medium for 2–3 days for subsequent analysis.

### Construction of expression vectors

All expression vectors were constructed on pcDNA3.1(+)/myc-His vector (Invitrogen) unless otherwise specified. Plasmids containing PAR1 and CD4-HA-AATN/KKTN cDNAs were kindly provided by Dr.s Hua-Wen Fu and Shaun R. Coughlin and Min Li, respectively^37^. Expression constructs encoding mFc, EMR2-WT-mFc, EMR2-S518A-mFc, EMR2-WT-myc, EMR2-S518A-myc and the EMR2-PAR1 chimeric proteins have been described previously^37^. Constructs encoding the chimeric EMR2-CD4 proteins were made by cloning the CD4-HA-AATN/KKTN fragments immediately downstream of the respective EMR2 NTF cDNAs via the *Bgl* II and *Apa* I sites. The individual N-glycosylation site-specific mutants (N298Q, N347Q, N354Q, N456Q and N460Q) of EMR2-WT-mFc were generated in a two-step PCR procedure by using EMR2-WT-mFc cDNA as template and site-specific mutant primers. N-terminal tagged EGFP-encoding constructs containing designated EMR2-NTF peptides, including H1 (aa 265–294), AH (aa 328–357) and control (aa 391–420), were made by ligating the cDNA fragments of respective peptides into pEGFP-N1 vector (Clontech, Hampshire, UK) via *EcoR*I site. The AH (mutant)-EGFP containing three site-directed point mutations (L333D, L341E and L344E) in AH was generated in a two-step PCR procedure by using AH-EGFP cDNA as the template.

### Protein secondary structure prediction and biochemical analysis

Protein secondary structure and amphipathic α-helix prediction was done using AmphipaSeeK v1.3.5 program as described^39,40^. Helical-wheel projections of peptides were predicted using the HeliQuest server as described^41^. Cell lysates were collected by cell lysis in modified RIPA lysis buffer (20 mM TrisHCl pH 7.4, 5 mM MgCl_2_, 100 mM NaCl, 0.5% NP-40 and 1X Complete Protease Inhibitors) supplemented with 1 mM sodium orthovanadate, 1 mM AEBSF and 5 mM Levamisole. SDS-PAGE and western blot analysis were carried out using standard procedures as described previously^37,42^. For de-glycosylation experiments, protein samples were first denatured in denaturing buffer (0.5% SDS, 1% β-mercaptoethanol) at 85 °C for 3 min. Denatured samples were incubated with either endoglycosidase H (1 U) alone or different combinations of PNGase F (1 U) and neuraminidase (1.0 mU) in 20 mM sodium phosphate buffer, pH 6.8 at room temperatures for 1–4 h, then at 37 °C overnight as described previously^43^.

Perfluorooctanoic-acid (PFO) and thrombin treatment of cells was performed as described previously^37^. Briefly, cells were incubated in ~500 μl of thrombin digestion solution (10 mM HEPES-Opti-MEM) in the presence or absence of thrombin (200 nM, 23.5 U) for 30 min at 37 °C before the supernatants and cells were collected by centrifugation at 500 g for 5 min. For PFO treatment, cells were incubated for 10 min on ice in PFO-containing PBS buffer (PFO solution). The supernatant was collected following centrifugation at 13,000 rpm at 4 °C for 15 min and the pellet was dissolved in modified RIPA lysis buffer.

Triton X-114 phase separation of proteins was performed as described previously^44^. Briefly, total cell lysate and PFO-treated supernatant were extracted by adding 0.1 ml of 2% (v/v) pre-condensed Triton X-114 solution (10 mM Tris-HCl pH 7.4, 150 mM NaCl and 2% Triton-X 114) for 1 h on ice. Triton-X 114 detergent-extracted samples (200 μl) were then layered on to a 300 μl 6% (w/v) sucrose cushion (10 mM Tris-HCl pH 7.4, 150 mM NaCl, 0.06% Triton-X 114 and 6% sucrose), incubated at 30 °C for 3 min and finally centrifuged at 300 g for 3 min. After centrifugation, the bottom detergent phase was collected (DP1) and the upper aqueous phase was re-extracted with 1% Triton X-114 and subjected to a second separation through the same sucrose cushion. After a second centrifugation step, the bottom detergent phase (DP2) and the upper aqueous phase (AP) were collected for analysis.

The fractionation of crude membrane and cytosol was performed as described previously^45^. Briefly, cells were resuspended in the 25 mM HEPES buffer, pH7.2 containing 1X protease inhibitor and swollen on ice for 60 min. Cells were lysed by three freeze-thaw cycles and centrifuged at 1,000 g for 10 min at 4 °C to collect the nuclei + pellets. The supernatant was collected and centrifuged for 60 min at 100,000 g (35,000 rpm; rotor SW50.1; Beckmann) to give a crude membrane pellet and cytosol supernatant. For further extraction of peripheral proteins^46^, the crude membrane pellets were treated with 1 M NaCl or 100 mM Na_2_CO_3_, pH 11 overnight and then ultra-centrifuged at 100,000 g for 60 min at 4 °C.

### Flow cytometry

Transiently transfected cells were harvested and fixed in 2% paraformaldehyde/PBS at 4 °C for 20 min. Cells were then blocked for 1 hr in ice-cold blocking buffer (1% BSA/5% serum of 2° Ab/PBS) with or without 0.1% saponin. Cells were subsequently incubated with the indicated primary antibody diluted in blocking buffer for 1 hr before washing. Cells were incubated for 1 hr with fluorochrome-conjugated 2^nd^ Ab in blocking buffer (1:200) and then washed three times by cold PBS and subjected to analysis by FACScan flow cytometer (BD Biosciences).

### Confocal immunofluorescent microscopy

Transiently transfected cells cultured on coverslips (BD BioCoat™ Poly-D-Lysine, BD Biosciences) were fixed in 2% paraformaldehyde/PBS at 4 °C for 30 min. Cells were then blocked for 1 hr in ice-cold blocking buffer (1% BSA/5% serum of 2° Ab/PBS) with or without 0.1% saponin. Cells were incubated with 2A1 mAb (5 ug/ml) for 1 hr, followed by Alexa-Fluor 594-conjugated anti-mouse IgG (1:200; Invitrogen) for 1 hr on ice with extensive washes in between by ice-cold blocking buffer. Coverslips were mounted onto slides with anti-fade mounting medium (ProLong® Gold Antifade reagent with DAPI, Invitrogen). Fluorescence images were taken at 1000-fold magnification using a confocal microscope (Zeiss LSM 510 META).

## Results

### Detection of differential N-glycosylation of the membrane-associated EMR2-NTF

**Figure 1:**
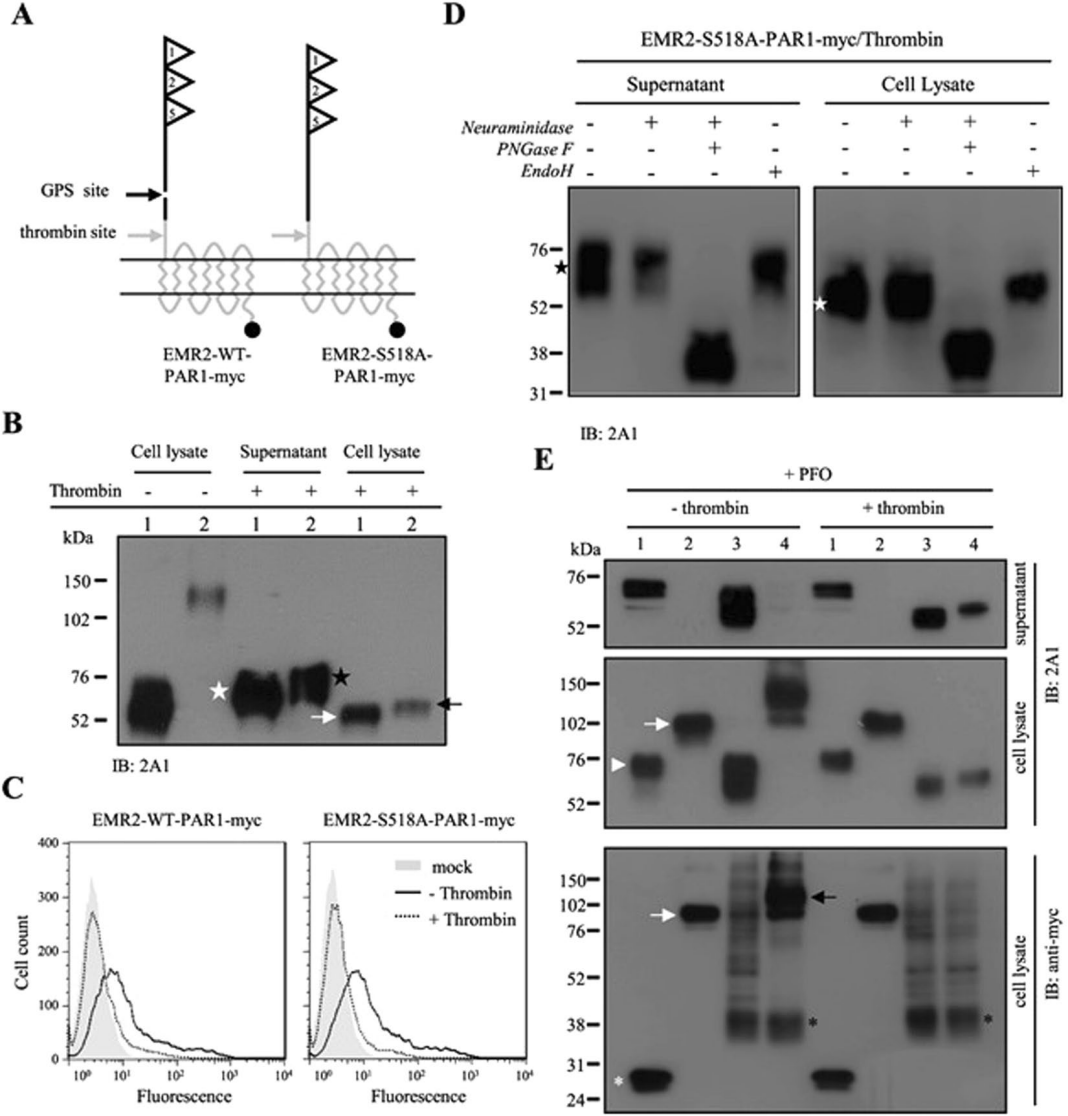
Glycosylation-dependent membrane-association of EMR2-NTF. (**A**) Schematic representation of the chimeric EMR2-WT-PAR1-myc and EMR2-S518A-PAR1-myc proteins. The EGF-like 1, 2 and 5 motifs of EMR2 protein are represented by three triangles. The GPS and thrombin cleavage sites are indicated by a black and a gray arrow, respectively. The myc tag is represented by a black circle. (**B**) WB analysis of HEK293T cells expressing EMR2-WT-PAR1-myc (#1) and EMR2-S518A-PAR1-myc (#2) treated with or without thrombin. Note the sizes of the thrombin-released NTFs in supernatant (white and black stars) are bigger than the NTFs remained in cell lysate (white and black arrows). (**C**) Flow cytometry analysis of the surface EMR2 expression of transfected HEK293T cells treated with (dash line) or without (black line) thrombin. Mock transfected HEK293T cells were negative controls. (**D**) WB analysis of supernatant and cell lysate of thrombin-digested HEK293T cells expressing EMR2-S518A-PAR1-myc treated with indicated de-glycosylation enzymes. The heterodimeric and membrane-associated EMR2-NTF molecules are indicated by a black star and a white star, respectively. (**E**) WB analysis of supernatant and cell lysate of PFO-treated HEK293T cells. Transfected cells expressing EMR2-WT-myc (#1), EMR2-S518A-myc (#2), EMR2-WT-PAR1-myc (#3) and EMR2-S518A-PAR1-myc (#4) were treated with or without thrombin first, washed and incubated with 0.2% PFO. Supernatant and cell lysate were then collected and analyzed by WB analysis using 2A1 and anti-myc mAbs as indicated. The white arrowhead and asterisk indicate the NTF and CTF of EMR2-WT-myc respectively, whereas the white arrows represent the EMR2-S518A-myc. The black arrow indicates the uncleaved EMR2-S518A-PAR1-myc protein, while the black asterisk indicates the cleaved PAR1 fragment following thrombin digestion.

In order to discriminate the membrane-associated and hetero-dimeric NTFs of aGPCRs^36,37^, we analyzed two previously-described chimeric EMR2 receptors (EMR2-WT-PAR1-myc and EMR2-S518A-PAR1-myc) containing the EMR2-NTF fused with the protease-activated receptor 1 (PAR1) in which the GPS site is located upstream of the thrombin site (Fig. 1A). Consistent with earlier results, the EMR2-WT-PAR1-myc protein is fully processed by GPS cleavage while the GPS-deficient EMR2-S518A-PAR1-myc molecule is uncleaved (Fig. 1B). Thrombin digestion of the chimeric receptors is shown to be fully competent as evidenced by the complete loss of the ~120–140 kDa EMR2-S518A-PAR1-myc band in cell lysate and the concurrent appearance of ~65–80 kDa EMR2-NTF in the supernatant (Fig. 1B). Interestingly, thrombin treatment of cells expressing EMR2-WT-PAR1-myc also sheds significant amounts of EMR2-NTF into supernatant, indicating that the majority of EMR2-NTF forms a hetero-dimeric complex with the CTF.

Nevertheless, a low but significant level of EMR2-NTF on the membrane was readily detected following thrombin treatment of transfected cells (Fig. 1B,C). This result confirms that a small fraction of EMR2-NTF is indeed able to associate independently on the membrane as otherwise thrombin digestion would have removed all surface NTFs. Intriguingly, an apparent size difference was noted between the thrombin-released hetero-dimeric NTF and the one remained on the membrane. More precisely, the hetero-dimeric NTF is larger than the membrane-associated NTF (Fig. 1B). We further characterized the two NTF forms using de-glycosylation enzymes and found them both to be neuraminidase- and Endo H-resistant, but sensitive to PNGase F (Fig. 1D). Importantly, the fully de-glycosylated core proteins of both NTF forms are the same size. These results indicate that the hetero-dimeric and membrane-associated NTFs have all completed post-translational glycosylation, but likely contained different numbers/composition of complex N-glycans (Fig. 1D).

To further verify the independent membrane association of EMR2-NTF, cells were treated with PFO, a weak surfactant capable of dissociating membrane-associated NTF without affecting aGPCR NTF-CTF complex^37^. Consistent with earlier results, 0.2% PFO efficiently removed the membrane-associated EMR2-NTF from cells expressing EMR2-WT molecules, but not those expressing EMR2-S518A receptors in the absence of thrombin (Fig. 1E). Importantly, the membrane-associated NTFs of thrombin-cleaved chimeric EMR2-PAR1 receptors were readily dissociated from cell membrane by 0.2% PFO (Fig. 1E). Taken together, we conclude that a small fraction of EMR2-NTF seems to undergo a different pattern of N-glycosylation compared to the hetero-dimeric NTF, enabling it to associate on the membrane by itself.

### Biochemical characterization of the membrane-associated EMR2-NTF

**Figure 2:**
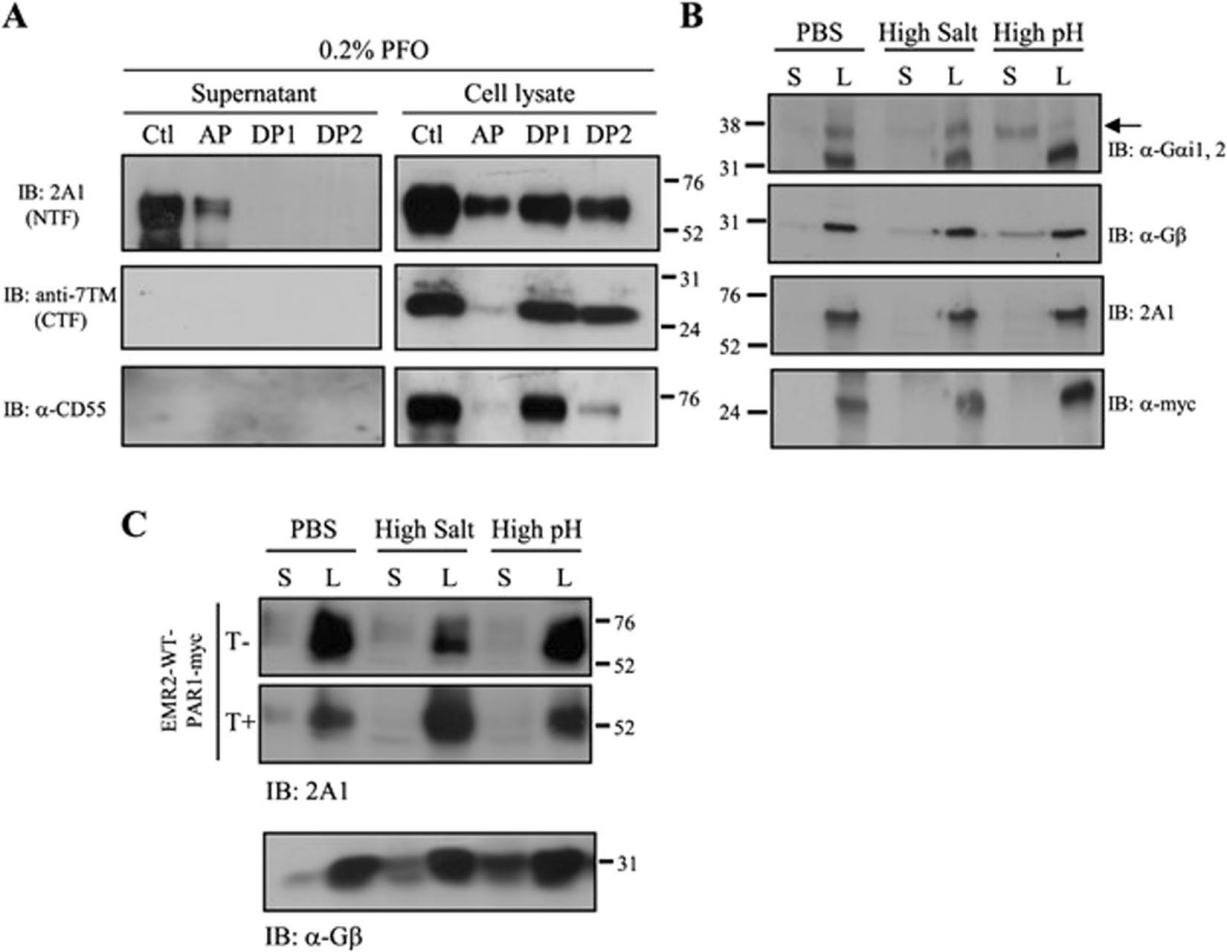
The hydrophilic characteristics of membrane-associated EMR2-NTF. (**A**) WB analysis of supernatant and cell lysate samples subjected to Triton X-114 phase separation. Supernatant and cell lysate were collected from PFO-treated CHO-K1 cells expressing EMR2-WT-myc. Ctl: input control protein samples before Triton X-114 phase separation; AP: aqueous phase; DP1 and 2: the first and second detergent phases, respectively. (**B**) WB analysis of membrane fraction of EMR2-WT-myc expressing cells after PBS, high salt (1 M NaCl) and high pH (100 mM Na_2_CO_3_, pH 11) treatment. S and L represent supernatant and cell pellets, respectively. The arrow indicates the specific Gαi1,2 protein band. (**C**) WB analysis of membrane fraction of thrombin-treated (T+) or untreated (T−) EMR2-WT-PAR1-myc expressing cells, followed by PBS, high salt (1 M NaCl) and high pH (100 mM Na_2_CO_3_, pH 11) treatment. Blots were probed with indicated mAbs.

Proteins without any TM domain may associate with membrane as a result of post-translational modification by specific lipid moieties. To delineate the biochemical characteristics of membrane-associated EMR2-NTF, we next analyzed its hydrophobicity by performing Triton-X 114 phase separation by which the hydrophilic proteins were kept in the aqueous phase (AP) whereas the hydrophobic proteins were segregated in the detergent phase (DP). As shown, the PFO-solubilized (membrane-anchored) EMR2-NTF was predominantly found in AP, while the remaining hetero-dimeric NTF was detected mostly in DP (Fig. 2A). As expected, the EMR2-CTF and GPI-linked CD55 were not solubilized by PFO and remained mainly in DP (Fig. 2A).

We next tested whether the membrane-associated EMR2-NTF can be extracted from cell membrane by 1 M NaCl (high salt) or 100 mM Na_2_CO_3_, pH 11 (high pH), conditions usually used to dissociate some peripheral membrane proteins^46^. As shown, the EMR2-NTF was not solubilized by high salt or high pH treatment of cells expressing EMR2-WT-myc (Fig. 2B). By contrast, the lipid-modified G_αi1,2_ and G_β_ peripheral membrane proteins were readily dissociated from membrane by the same treatments (Fig. 2B). This data confirms that the majority of the EMR2-WT-myc is a tightly-bound NTF-CTF receptor complex. Interestingly, high salt and high pH treatments still failed to dissociate the NTF of EMR2-WT-PAR1-myc protein from membrane even after thrombin digestion (Fig. 2C). Hence, we conclude that the membrane-associated EMR2-NTF is mostly hydrophilic in nature and is not likely modified by lipid moieties (Fig. 2B,C).

### Membrane-association of EMR2-NTF takes place in post-ER compartments during receptor trafficking

**Figure 3:**
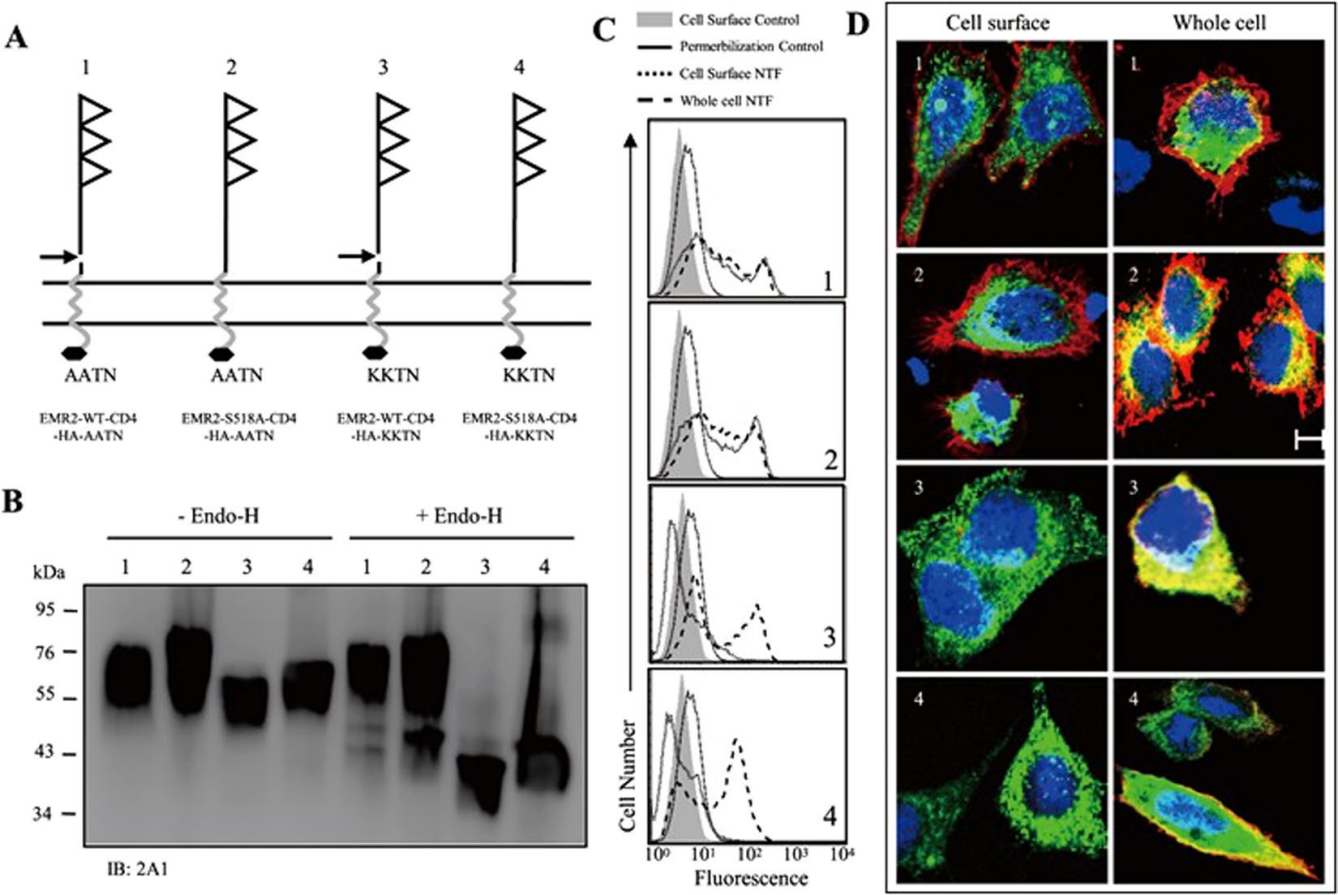
Membrane-association of EMR2-NTF occurs in post-ER compartments. (**A**) Schematic representation of the chimeric EMR2-CD4 fusion proteins including EMR2-WT-CD4-HA-AATN (1), EMR2-S518A-CD4-HA-AATN (2), EMR2-WT-CD4 -HA-KKTN (3) and EMR2-S518A-CD4-HA-KKTN (4). The CD4 TM region and HA tag are represented by a single gray zig-zag line and a black hexagon, respectively. KKTN and AATN indicate the C-terminal ER retention signal peptide and control peptide, respectively. (**B**) WB analysis of total cell lysates of CHO-K1 cells expressing the respective chimeric EMR2-CD4 fusion proteins as indicated by the numbers in (**A**). Lysates were digested without (left panel) or with (right panel) Endo-H. The blot was probed with the 2A1 mAb. (**C**) FACS analysis of EMR2-NTF expression levels on cell surface and permeabilized whole cell of CKO-K1 cells expressing the respective chimeric EMR2-CD4 fusion proteins as indicated by the numbers in (**A**). (**D**) Confocal immunofluorescence analysis of EMR2-NTF expression patterns on cell surface (left panel) and whole cell (right panel) of CKO-K1 cells expressing the respective chimeric EMR2-CD4 fusion proteins as indicated by the numbers in (**A**). The red color represents EMR2-NTF stained by 2A1 mAb followed by goat anti-mFc IgG-Alexa Flour 647. The green color represents the ER marker expressed by the transfected pCMV-myc-ER-GFP plasmid, while the blue color indicates the nucleus stained by DAPI. Scale: 10 μm.

The finding that both membrane-anchored and hetero-dimeric NTFs have all completed post-translational glycosylation prompted us to ask when and where are the two NTF forms generated during receptor maturation and trafficking? To this end, chimeric EMR2-CD4 fusion proteins, EMR2-WT-CD4-HA-AATN or -KKTN and EMR2-S518A-CD4-HA-AATN or -KKTN, containing the EMR2-NTF fused to the CD4-TM region which terminated with the ER retention signal KKTN peptide or the control AATN sequence were generated (Fig. 3A)^47^. Due to the presence of the ER retention signal, the KKTN-containing EMR2-CD4 fusion proteins are expected to remain in the ER compartment. Hence, one would expect to detect membrane-anchored EMR2-NTF on cell surface and the GPS-cleaved KKTN-containing CTF at ER if the dissociation of NTF and CTF occurs soon after GPS proteolysis. On the other hand, if the NTFs and CTFs of the KKTN-containing chimeric EMR2-CD4 fusion proteins showed a similar ER-restricted expression pattern, it is unlikely that the separation of the membrane-anchored NTF and CTF takes place within ER. As shown, the NTFs of KKTN-containing fusion proteins are smaller and Endo H-sensitive compared to those of the AATN-containing counterparts as a result of ER retention (Fig. 3B). Importantly, FACS and confocal immunofluorescence analyses both showed that the NTFs of the KKTN-containing fusion proteins remained within ER, unlike the cell surface expression of the control AATN-containing receptors (Fig. 3C,D). These results suggest that the membrane association of NTF likely takes place in post-ER compartments, probably in the Golgi or on the membrane.

### Membrane-association of EMR2-NTF is regulated by site-specific N-glycosylation

**Figure 4:**
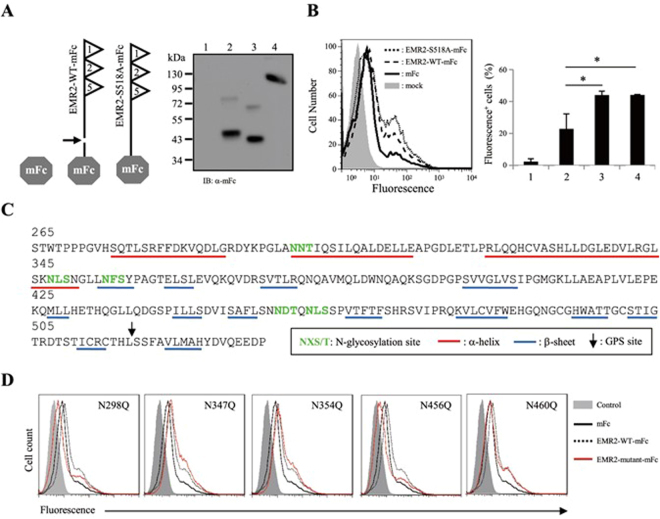
Membrane-association of EMR2-NTF is regulated by site-specific N-glycosylation. (**A**) Left panel, schematic representation of the chimeric EMR2-mFc fusion proteins. The GPS-cleavage site and the mFc domain are indicated by a black arrow and a gray hexagon, respectively. Right panel, WB analysis of the mFc (lane 2), EMR2-WT-mFc (lane 3) and EMR2-S518A-mFc (lane 4) fusion proteins in the supernatant of transfected CHO-K1 cells. Lane 1 contains the control sample from mock-transfected cells. (**B**) Flow cytometry analysis of the surface levels of the mFc-fusion proteins by the FITC-conjugated goat anti-mFc mAb. The graph shows the percentage of FL1^+^ cells. Samples include mock control CHO-K1 cells (lane 1) and cells expressing mFc (lane 2), EMR2-WT-mFc (lane 3) and EMR2-S518A-mFc (lane 4). (n = 3, mean ± SD; *p < 0.05). (**C**) The secondary structure prediction of the EMR2-GAIN domain (aa 265–534 based on the full-length sequence of the largest EMR2 isoform). The 5 potential N-glycosylation sites (NXS/T) are highlighted in green. The α-helix and β-sheet structures are underlined by red and blue lines, respectively. The GPS cleavage site is indicated by a black arrow. (**D**) Flow cytometry analysis of the surface mFc-fusion protein levels of cells expressing various N-glycosylation site mutants of EMR2-mFc as indicated.

To investigate the role of N-glycosylation in the association of EMR2-NTF with membrane, we analyzed the chimeric EMR2-mFc fusion proteins (EMR2-WT-mFc and EMR2-S518A-mFc) containing the EMR2-NTF conjugated to the mFc fragment (Fig. 4A). Interestingly, while the majority of the EMR2-mFc fusion proteins were detected in the supernatant as expected, FACS analysis revealed a low but significant level of the molecule on the cell surface (Fig. 4A,B). EMR2-NTF contains a total of five potential N-glycosylation sites in the GAIN domain. Hence, five individual N-glycosylation site point mutants of EMR2-WT-mFc molecules were generated (Fig. 4C). Interestingly, two mutants (N347Q and N354Q) displayed a higher surface level, while another two mutants (N298Q and N456Q) showed a lower surface level when compared to the WT molecule (Fig. 4D). By contrast, the fifth mutant (N460Q) was expressed at the same level as the WT molecule (Fig. 4D). These results indicate that the membrane-association ability of EMR2-NTF is regulated in part by site-specific N-glycosylation.

### An amphipathic α-helix in EMR2-NTF is a potential in-plane membrane anchor

**Figure 5:**
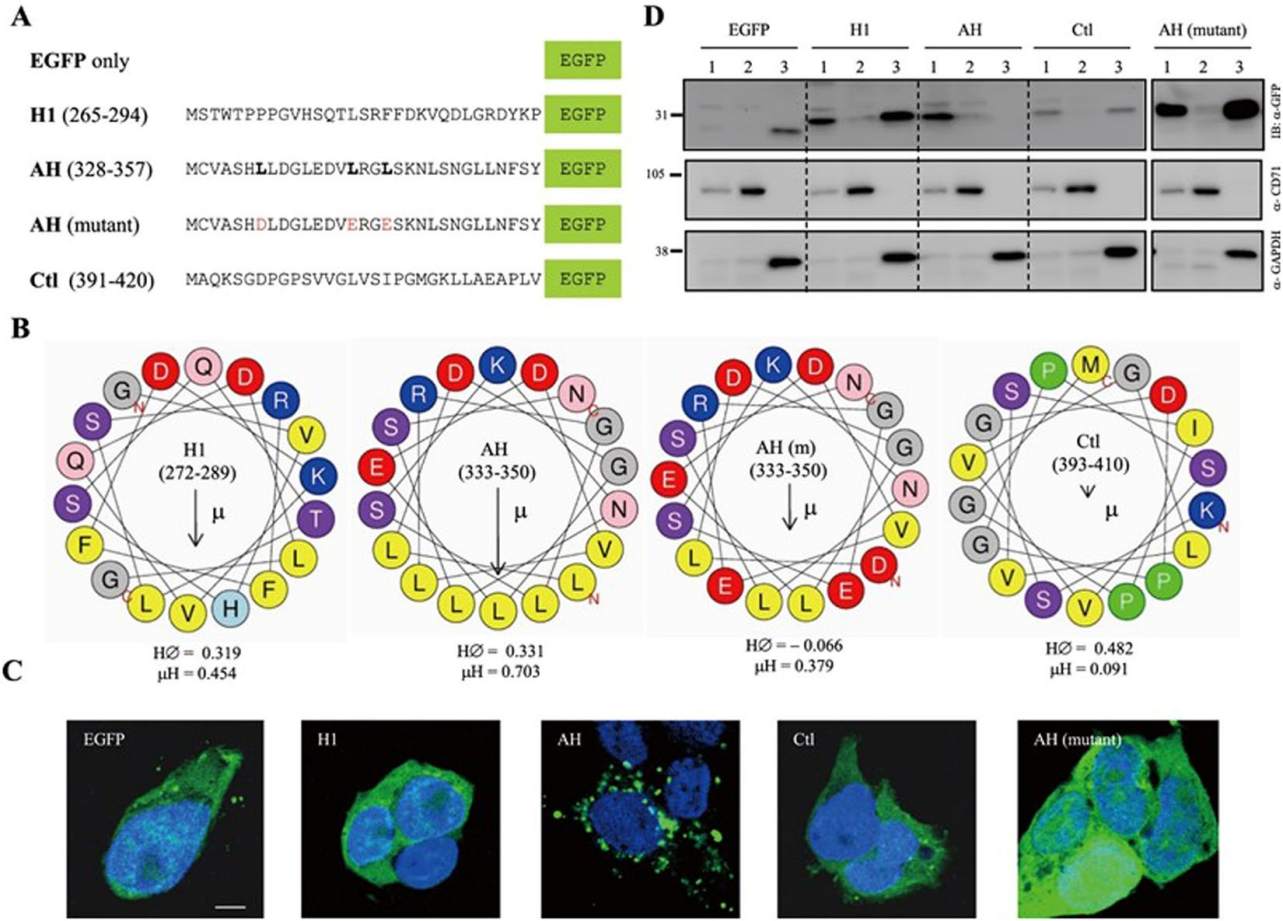
Identification of an amphipathic α-helix in EMR2-NTF as an in-plane membrane anchor. (**A**) Schematic representation of the N-terminal tagged EGFP-fusion proteins containing the H1 (aa 265–294), AH (aa 328–357), AH mutant (AH with L333D, L341E and L344E point mutations) and control (Ctl) peptides (aa 391–420) of EMR2-NTF. The EGFP-only is a negative control. (**B**) Helical-wheel projections of the corresponding peptides as indicated. The color codes for conserved residues are indicated below: yellow, hydrophobic; purple, serine and threonine; blue, basic; red, acidic; grey, other residues. The predicted Hydrophobicity (H∅) and Hydrophobic moment (μ) are shown below each helical-wheel. (**C**) Confocal fluorescence staining of transfected H293T cells expressing the EGFP-only, H1-EGFP, AH-EGFP, Ctl-EGFP and AH-mutant-EGFP. Blue and green colors indicate the nucleus and the EGFP protein, respectively. Scale: 5 μm. (**D**) WB analysis of the cell-fractionation samples of transfected cells expressing the EGFP-only, H1-EGFP, AH-EGFP, Ctl-EGFP and AH-mutant-EGFP. Cells were divided into (1) cell pellet + nucleus, (2) membrane and (3) cytosol fractions, respectively. CD71 and GADPH are used as the specific markers of the membrane and cytosol fractions, respectively.

The aforementioned results strongly suggested the presence of a possible membrane-interacting motif in EMR2-NTF. To investigate the putative membrane-interacting sequence and to explain why the N347Q and N354Q mutants could enhance the membrane association of EMR2-NTF, the secondary structure of the EMR2 GAIN domain was analyzed. Different from what was identified in the GAIN domains of Latrophilin-1/ADGRL1 and BAI 3/ADGRB3, 3 α-helixes and 13 β-strands were predicted (Fig. 4C). Interestingly, when analyzed by the AmphipaSeeK prediction program for the presence of possible in-plane membrane anchors, an amphipathic helix (AH) (_333_LLDGLEDVLRGLSKNLSN_350_) with very high amphipathic hydrophobicity score was found in the 3rd α-helix (Fig. 5B)^39,40^. More importantly, the N^347^ and N^354^ residues are located within or close to this AH. We hence investigated the membrane-association ability of this AH. To this end, expression constructs expressing EGFP fusion proteins N-terminally tagged with distinct peptide sequences were generated (Fig. 5A). As such, AH-EGFP contains residues 328–357 of EMR2-NTF that includes the AH region, and H1-EGFP contains residues 265–294 of EMR2-NTF that includes the first α-helix region. The AH mutant-EGFP contains the AH peptide with 3 point mutations (L333D, L341E and L344E), which are predicted to reduce the amphipathicity of the peptide dramatically. The Ctl-EGFP contains residues 391–420 and is used as a negative control (Fig. 5A,B).

Confocal fluorescence staining showed a distinct pattern of intracellular punctuated membrane patches for the AH-EGFP protein. By contrast, a homogenous cytoplasmic pattern was observed for all other EGFP-fusion proteins and the control EGFP-only protein (Fig. 5C). Similarly, the AH-EGFP protein was detected predominantly in the membrane but not the cytosolic fraction in the cellular fractionation assay (Fig. 5D). As expected, H1-EGFP and Ctl-EGFP fusion proteins and the control EGFP-only protein were detected mostly in the cytosolic fraction. Importantly, the AH mutant-EGFP protein behaved exactly like the H1-EGFP and Ctl-EGFP proteins, suggesting that the three hydrophobic leucine residues and hence the amphipathicity of the peptide are critical for the membrane-interacting capacity of the AH peptide (Fig. 5D). In summary, the AH motif of the EMR2-NTF is a potential in-plane membrane-anchor mainly due to its strong amphipathicity. Due to the close proximity of the N^347^ and N^354^ residues to the AH motif, the lack of N-glycosylation likely makes the AH motifs of the N347Q and N354Q mutants more exposed and accessible hence promoting the interaction of the EMR2-NTF proteins with the membrane.

## Discussion

Recent advances have indicated that the aGPCR-mediated activity is critically dependent on the strength and mode of the NTF-CTF interaction. Ligand binding to the NTF might induce its shedding from and the subsequent activation of the CTF by the Stachel-dependent mechanism as seen in the tethered agonist model^25,27^. Alternatively, ligand binding might enhance or reduce the strength/affinity of the NTF-CTF interaction to fine tune the signaling activities in a GPS proteolysis-independent and Stachel-independent mechanism^30,32–34^.

In this report, we investigated the molecular characteristics of a novel NTF form that remains associated on the cell surface by itself. Indeed, a small percentage of the EMR2-NTF was found to associate independently on the membrane even though the majority of the EMR2-NTF was coupled with the CTF as a dual-subunit complex (Fig. 1A–C). Most interestingly, we found that the membrane-associated EMR2-NTF is an amphipathic molecule modified by differential N-glycosylation but not any lipid moiety (Figs 1D,E and 2). The membrane association of EMR2-NTF seems to take place in post-ER compartments during receptor trafficking (Fig. 3). This result suggests that the membrane association of EMR2-NTF is tightly regulated by post-translational processing. Indeed, site-specific N-glycosylation in the GAIN domain was identified to play a role in modulating the membrane association of the EMR2-NTF (Fig. 4). More importantly, the two N-glycosylation sites important for EMR2-NTF membrane association are located near a novel amphipathic α-helix that seems to function as an in-plane membrane anchor (Fig. 5). Hence, it is concluded that the membrane-associated EMR2-NTF form likely used the amphipathic α-helix in the GAIN domain as a membrane-anchoring segment to associate in-plane with the membrane. Local site-specific N-glycosylation in turn modulated the exposure and accessibility of the amphipathic α-helix and hence the membrane association of the EMR2-NTF.

Interestingly, several but not all aGPCRs including human CD97, BAI2, mouse EMR4, and rat latrophilin 2, were found to contain a similar AH sequence in the GAIN domain when analyzed by the same AmphipaSeeK program (data not shown). Of interest, we found a AH fragment in rat latrophilin 2, but not rat latrophilin 1. Most importantly, among the 6 putative N-glycosylation sites identified in the GAIN domain, 2 are located near the AH fragment of rat latrophilin 2 (data not shown). This is similar to what we found in the AH fragment of EMR2, suggesting again a possible role of site-specific N-glycosylation in modulating the interaction of the amphipathic α-helix with the membrane. Nevertheless, although the idea that the amphipathic α-helix works as a potential membrane-anchor of the NTF is promising a direct evidence is still lacking and requires the support of more experimental results in the future.

The presence of the membrane-associated NTF is highly suggestive of an aGPCR activation mechanism different from the models described previously. Indeed, earlier studies of latrophilin-1 and EMR2 have shown that the membrane-associated NTF and the respective CTF were localized at different membrane regions/microdomains^36,37^. Ligand binding to the membrane-associated NTF promoted its translocation and interaction with the CTF, eventually leading to receptor activation and signalling^36^. Nevertheless, the fact that the membrane-associated NTF represents only a minor fraction of the aGPCR protomer implies that its function is probably to fine tune the overall activity of the aGPCR. In light of this, it is of interest to note that the membrane association of the EMR2-NTF is modulated in part by site-specific N-glycosylation in the GAIN domain.

We have previously shown that site-specific N-glycosylation is also involved in regulating the GPS autoproteolytic processing of CD97/ADGRE5, probably by adopting a non-cleavable conformation^48^. By analogy, it is possible that differential N-glycosylation in the GAIN domain may also alter the conformational restraint between the NTF and CTF to promote the membrane association of the NTF. Due to the fact that most aGPCRs are glycoproteins heavily decorated by N-glycosylation, this finding signifies the importance of N-glycosylation when considering the activation and function of aGPCRs. Nevertheless, in light of the novel allosteric antagonism of aGPCRs proposed recently^30^, we could not rule out the possibility that the membrane-associated NTF might not be dissociated completely from the CTF, but instead represent a subtype that is “stretched” on the membrane due to its glycosylation-dependent interaction of the amphipathic helix with the membrane. This idea is especially prudent if one considers the fact that the fully dissociated NTF that lacks the last β-strand would not have a stably folded GAIN structure and hence would collapse. Several aGPCRs have been shown to interact with specific cellular ligands, e.g. EMR2 and CD97 were known to interact with distinct glycosaminoglycans (GAGs) present on cell surface proteoglycans. It is hypothetically possible that the combined interaction of cellular ligands/GAGs and NTF as well as the amphipathic α-helix with the membrane might stabilize the “stretched” membrane-associated NTF.

Taken together, the membrane-associated EMR2-NTF was characterized as a novel NTF form with a differential N-glycosylation pattern and site-specific N-glycosylation near a putative amphipathic in-plane membrane anchor in the GAIN domain is critical in its ability to self-associate with cell membrane. Our results provide evidence of an alternative composition and activation mechanism of aGPCRs and additional insights into the functional complexity of this unusual GPCR family.

## Electronic supplementary material

Supplementary Information
