# Supplementary material for: Membrane-association of EMR2/ADGRE2-NTF is regulated by site-specific N-glycosylation
Source: Sci Rep. 2018 Mar 14;8:4532. doi: 10.1038/s41598-018-22849-x (PMC5852045; doi:10.1038/s41598-018-22849-x)
Supplement: Supplementary file 1 — Supplementary Information [file 41598_2018_22849_MOESM1_ESM.pdf]

# Membrane-association of EMR2/ADGRE2-NTF is regulated by site-specific N-glycosylation

Yi-Shu Huang[1,#,&], Nien-Yi Chiang[1,&], Gin-Wen Chang[1], Hsi-Hsien Lin[1,2,3]*

## Suppl. Figure 1

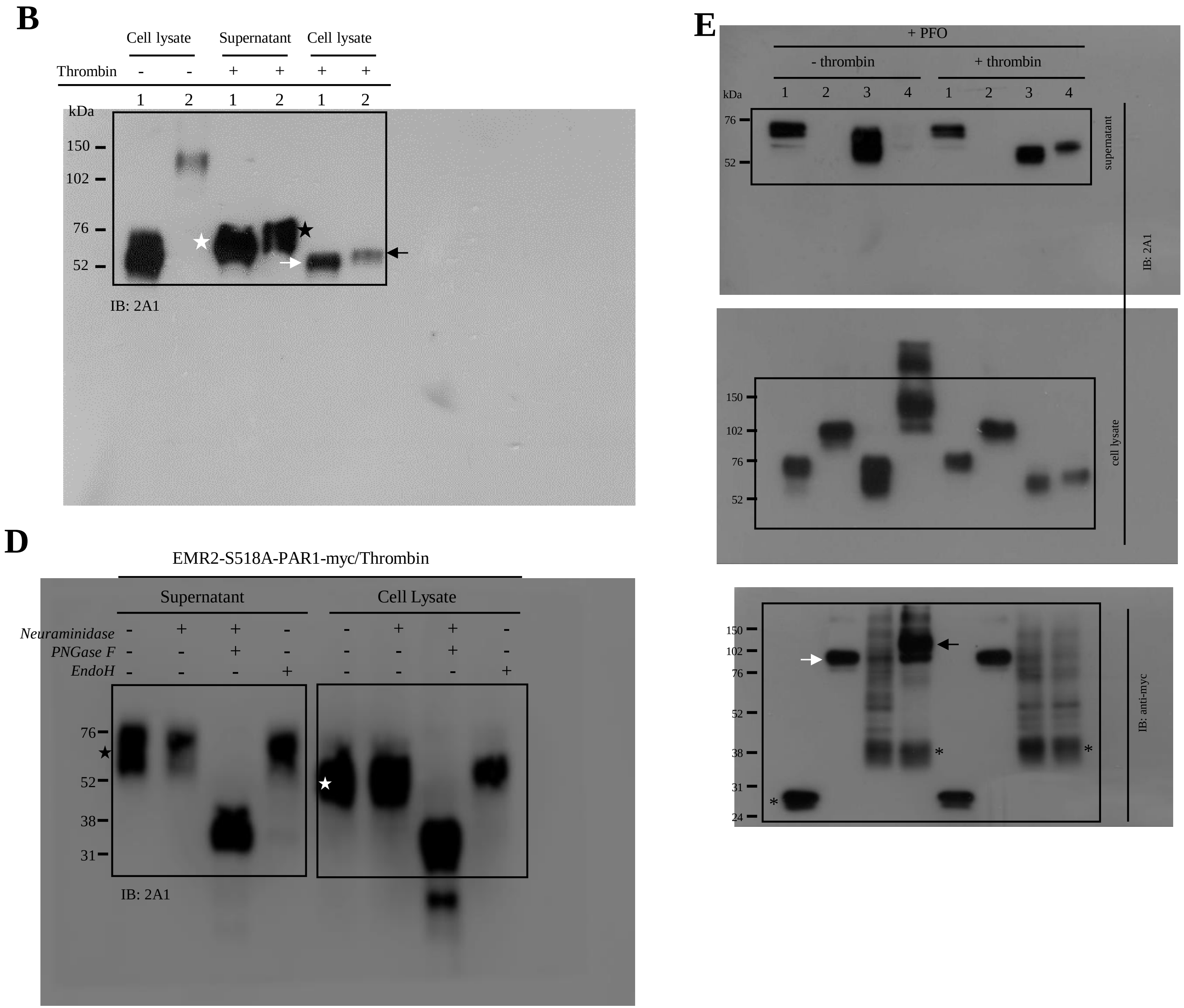

# Suppl. Figure 2

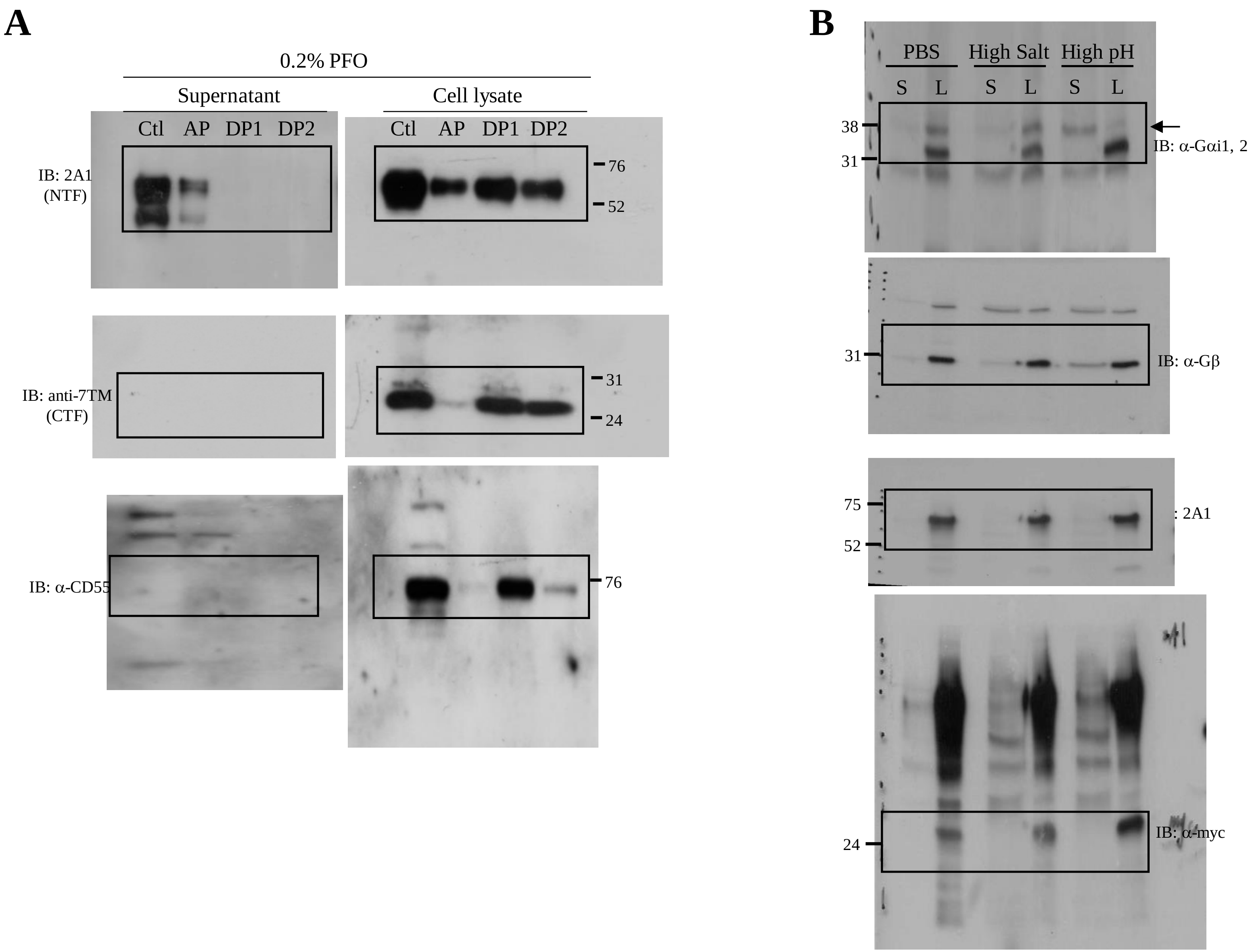

**Suppl. Figure 2**

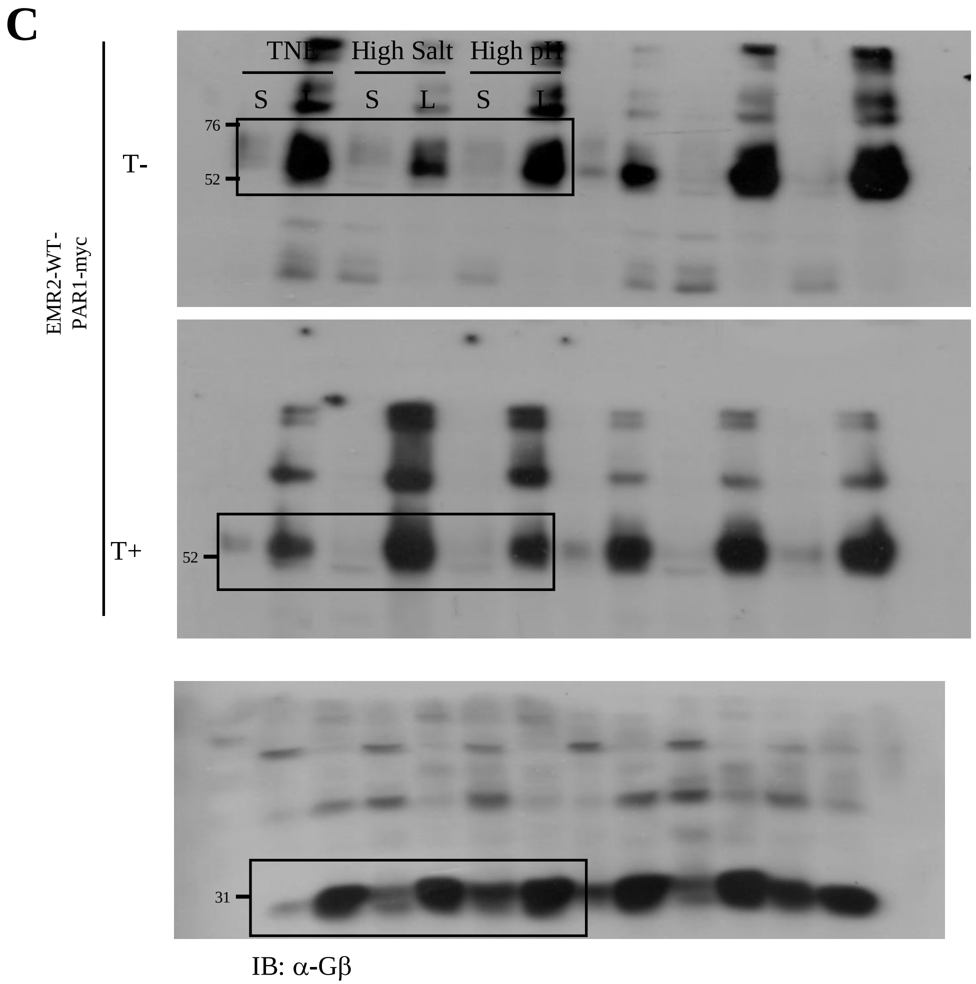

**Suppl. Figure 3**

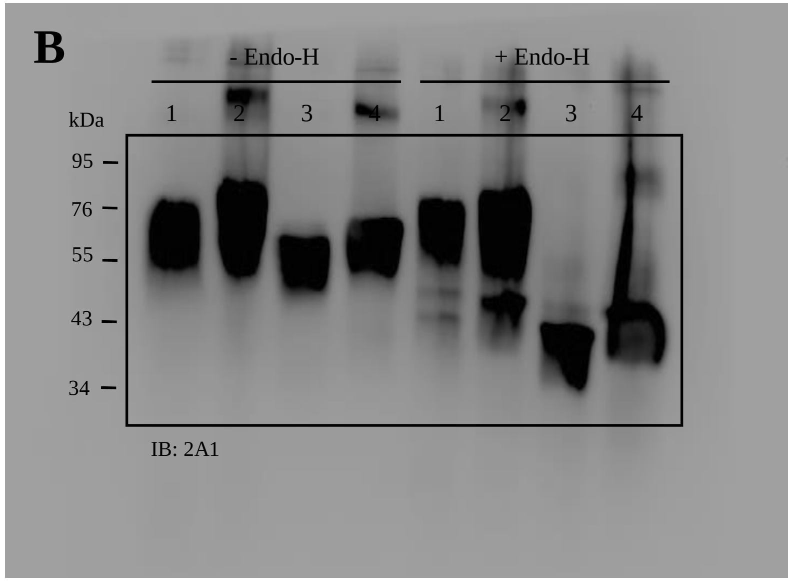

## Suppl. Figure 4

A

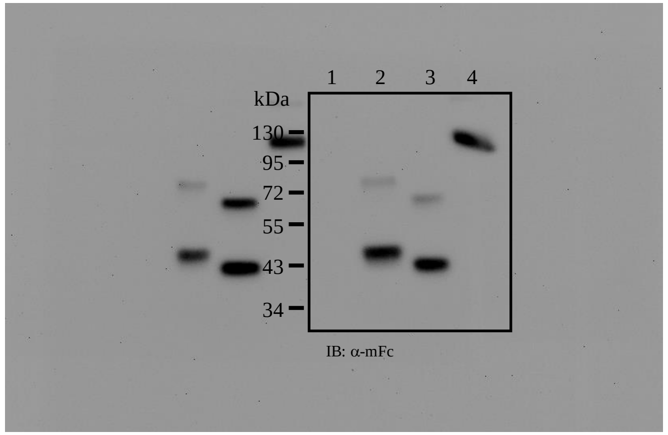

# Suppl. Figure 5

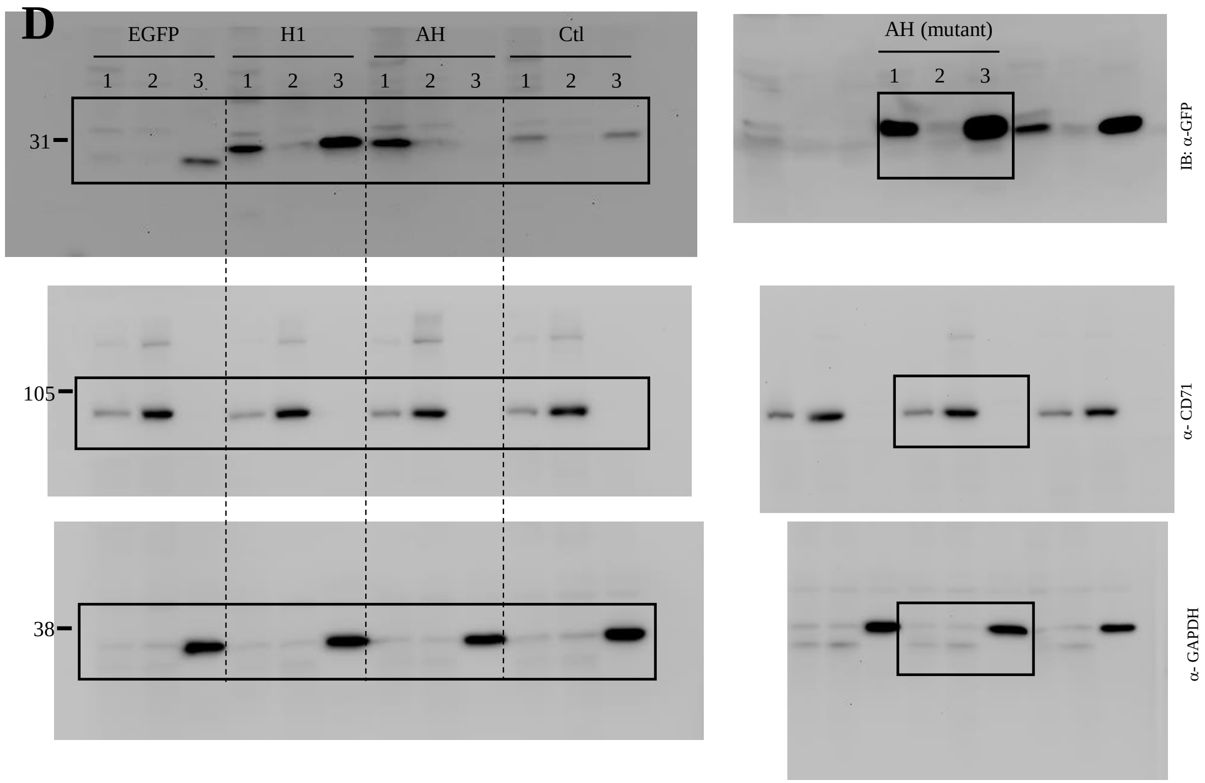

## Suppl. Figure 6

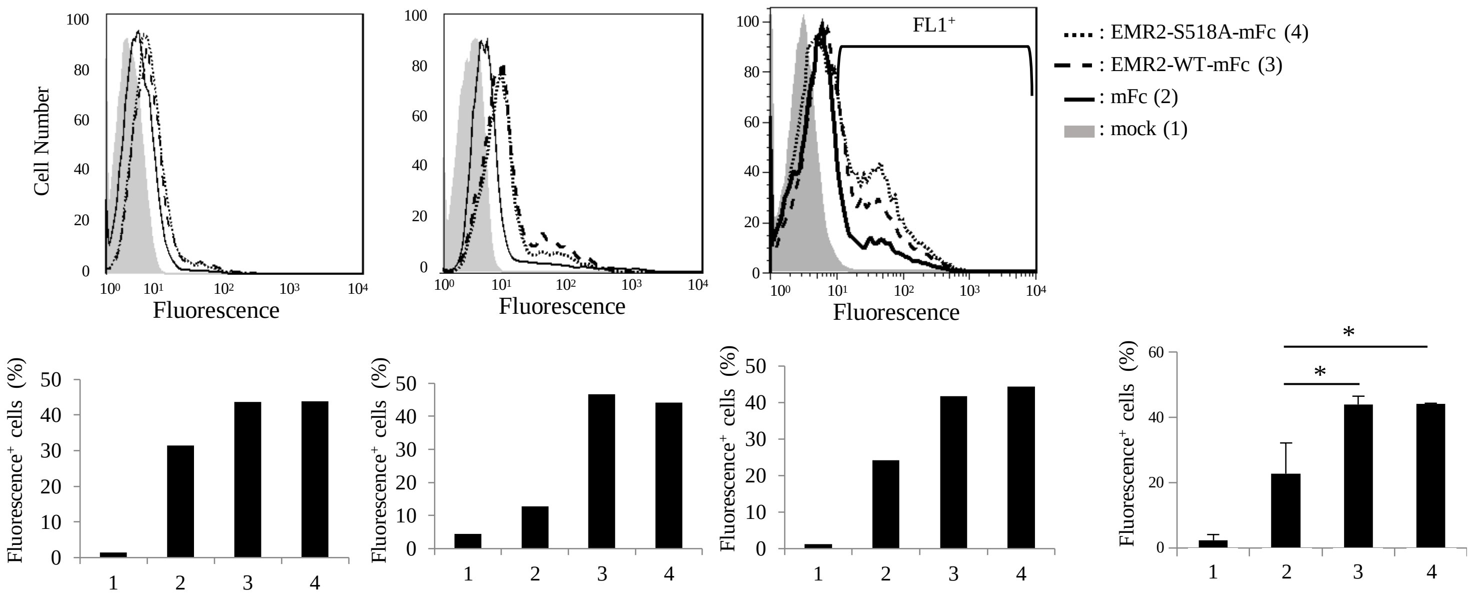


**Suppl. Fig. 6** Flow cytometry analysis of the surface levels of the mFc-fusion proteins by the FITC-conjugated goat anti-mFc mAb. The graph shows the percentage of $FL1^+$ cells. Samples include mock control CHO-K1 cells (lane 1) and cells expressing mFc (lane 2), EMR2-WT-mFc (lane 3) and EMR2-S518A-mFc (lane 4). The graph at the far right represents the combined results of three independent experiments (n=3, mean ± SD; *$p$< 0.05).

## Suppl. Figure 7

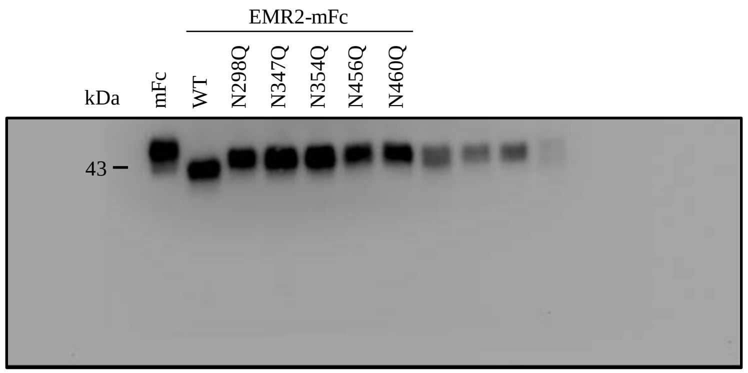


**Suppl. Fig. 7 Western blot analysis of the expression of mFc as well as WT and mutant EMR2-mFc molecules.** Supernatant of HEK293T cells transfected with the mFc expression constructs as indicated was collected and processed for the western blot analysis using the anti-mFc Ab. Each lane contained the same volume of supernatant.

## Suppl. Figure 8

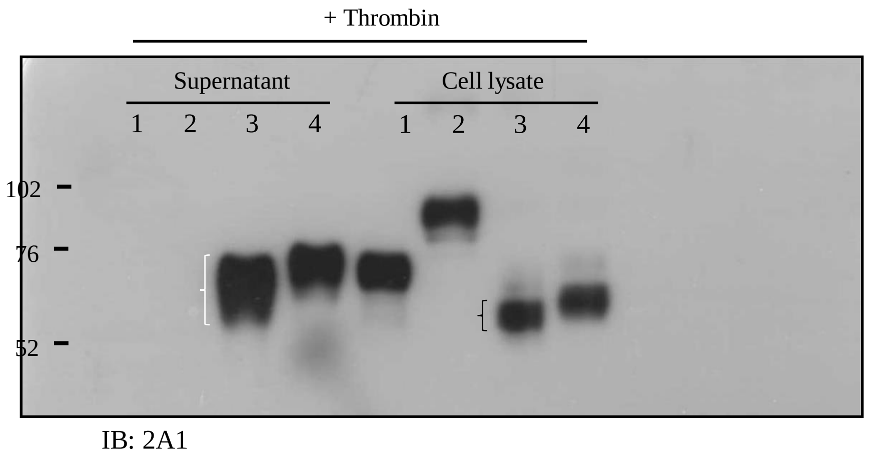


**Suppl. Fig. 8 Highly differential glycosylation of EMR2-PAR1 chimeric receptors.** WB analysis of supernatant and cell lysate of HEK293T cells expressing EMR2-WT-myc (lane 1), EMR2-S518A-myc (lane 2), EMR2-WT-PAR1-myc (lane 3), and EMR2-S518A-PAR1-myc (lane 4) treated with thrombin. Note the sizes of the thrombin-released NTF of EMR2-WT-PAR1-myc in supernatant (lane 3, white bracket) are bigger than the NTF remained in cell lysate (lane 3, black bracket). The same is true for the NTFs of EMR2-S518A-PAR1-myc (both lanes 4).

## SUPPL. FIGURE 9

Prediction of the AH sequence by the AmphipaSeeK v1.3.5

AmphipaSeeK v1.3.5- prediction of in-plane membrane anchoring segments -

AUHTOR : Nicolas Sapay, Institut de Biologie et Chimie de Proteines

UMR 5086 Centre National de la Recherche Scientifique - University Lyon 1

Lyon, France

NAME : AmphipaSeeK

VERSION : release 1.3.5

REFERENCE : Sapay,N., Guermeur,Y., Deleage,G. (2006) Prediction of in-plane membrane

anchors in monotopic proteins using a SVM classifier, BMC Bioinformatics, 7:255

USAGE :

>Nowhere|JohnDoe <--- sequence header

================

1 10

|........|.... <------ sequence position

APVVLSRRVLASAS <------ protein sequence

__AAAAAAA_____ <------ predicted membrane topology : 'A'=in-plane | '_'=non in-plane

chhhhhhhhhhhcc <------ predicted secondary structure (if available)

11224553210001 <------ amphipathy : from 0=low to 5=high amphipathy

based on the sequence average <muH>

EMR2

GAIN (250-529)+ GPS (479-529)

DMTFSTWTPPPGVHSQTLSRFFDKVQDLGRDYKPGLANNTIQSILQALDELLEAPGDLETLPRLQQHCV
ASHLLDGLEDVLRGLSKNLSNGLLNFSYPAGTELSLEVQKQVDRSVTLRQNQAVMQLDWNQAQKSGDPG
PSVVGLVSIPGMGKLLAEAPLVLEPEKQMLLHETHQGLLQDGSPILLSDVISAFLSNNDTQNLSSPVTF
TFSHRSVIPRQKVLCVFWEHGQNGCGHWATTGCSTIGTRDTSTICRCTHLSSFAVLMAHYDVQ

```
1        10         20         30         40         50         60         70         80
|........|.........|.........|.........|.........|.........|.........|.........|
DMTFSTWTPPPGVHSQTLSRFFDKVQDLGRDYKPGLANNTIQSILQALDELLEAPGDLETLPRLQQHCVASHLLDGLEDV
__________________________________________________________________________AAAAAA
ccceeecccccccccchhhhhhhhhhhhhhccccccccchhhhhhhhhhhhhhhccccccccchhhhhhhhhhhhhhhhhh
12222211111111233345555555444443332112334444444444433343322333334333221223344455555

81       90         100        110        120        130        140        150        160
|........|.........|.........|.........|.........|.........|.........|.........|
LRGLSKNLSNGLLNFSYPAGTELSLEVQKQVDRSVTLRQNQAVMQLDWNQAQKSGDPGPSVVGLVSIPGMGKLLAEAPLV
AAAAAA__________________________________________________________________________
hhhhhhhhhccxeeecccccccxxeehhhhhhhhheeexhhhhhhhhhhhhhhcccccccccceeeeeecccchhhhhhccce
55554443333332222221122323343444222222223221122222233333332222211122223332233211

161      170        180        190        200        210        220        230        240
|........|.........|.........|.........|.........|.........|.........|.........|
LEPEKQMLLHETHQGLLQDGSPILLSDVISAFLSNNDTQNLSSPVTFTFSHRSVIPRQKVLCVFWEHGQNGCGHWATTGC
________________________________________________________________________________
xccchhheehhhhhcccccccccceexxcccceexcccccccccccceeeeeccccccccceeeeeeeccccccccceeeeccc
22222211123233222332322222222332222111223322222222222211222111223333223322222222222

241      250        260        270        280        290        300        310        320
|........|.........|.........|.........|.........|.........|.........|.........|
STIGTRDTSTICRCTHLSSFAVLMAHYDVQEEDP
__________________________________
eeeccccccccceeecccccchhheeeeeecccccccc
2222233444423322222222211122111112 2
```

The N-glycosylation site is highlighted in yellow.

## CD97

DMTFSTWTPPPGVHSQTLSRFFDKVQDLGRDSKTSSAEVTIQNVIKLVDELMEAPGDVEALAPPVRHLI
ATQLLSNLEDIMRILAKSLPKGPFTYISPSNTELTLMIQERGDKNVTMGQSSARMKLNWAVAAGAEDPG
PAVAGILSIQNMTTLLANASLNLHSKKQAELEEIYESSIRGVQLRRLSAVNSIFLSHNNTKELNSPILF
AFSHLESSDGEAGRDPPAKDVMPGPRQELLCAFWKSDSDRGGHWATEGCQVLGSKNGSTTCQCSHLSSF
AILMAHYDVED

```
1         10        20        30        40        50        60        70        80
|........|.........|.........|.........|.........|.........|.........|.........|
DMTFSTWTPPPGVHSQTLSRFFDKVQDLGRDSKTSSAEVTIQNVIKLVDELMEAPGDVEALAPPVRHLIATQLLSNLEDI
_____________________________________________________________________________AAA
cccccccccccccchhhhhhhhhhhhcccccccccceehhhhhhhhhhhhhhcccccccxxcccccchhhhhhhhhhhhh
21111211111111233445555555444433333112233344555544434322222233443322221234445444

81        90        100       110       120       130       140       150       160
|........|.........|.........|.........|.........|.........|.........|.........|
MRILAKSLPKGPFTYISPSNTELTLMIQERGDKNVTMGQSSARMKLNWAVAAGAEDPGPAVAGILSIQNMTTLLANASLN
AAAAAAA_________________________________________________________________________
hhhhhhccccccceeeeccccchheeeeecccccceeeecccchhhhhhhhhhhccccccccceeeeeexxhhhhhhhhhhh
55555443333333221222121221222233333322332222222222222122222223222222222233322222232

161       170       180       190       200       210       220       230       240
|........|.........|.........|.........|.........|.........|.........|.........|
LHSKKQAELEEIYESSIRGVQLRRLSAVNSIFLSHNNTKELNSPILFAFSHLESSDGEAGRDPPAKDVMPGPRQELLCAF
________________________________________________________________________________
hchhhhhhhhhhhhhhhccxeehhhhccceeeeeccccccccccceeeeeecccccccccccccccccccccccchhhhhhh
11221122222224444422111122233222222222232332332222221122233232222122344332222212233

241       250       260       270       280       290       300       310       320
|........|.........|.........|.........|.........|.........|.........|.........|
WKSDSDRGGHWATEGCQVLGSKNGSTTCQCSHLSSFAILMAHYDVED
_______________________________________________
hhccccccccceeccceeeeeccccccceeeccchhhhhhhhhcccccc
32223333333343333222111223221122222122221112222333
```

hEMR3

DTTSSKTTEGRKELQKIVDKFESLLTNQTLWRTEGRQEISSTATTILRDVESKVLETALKDPEQ
KVLKIQNDSVAIETQAITDNCSEERKTFNLNVQMNSMDIRCSDIIQGDTQGPSAIAFISYSSLGNI
INATFFEEMDKKDQVYLNSQVVSAAIGPKRNVSLSKSVTLTFQHVKMTPSTKKVFCVYWKST
GQGSQWSRDGCFLIHVNKSHTMCNCSHLSSFAVLMALTSQ

```
1        10        20        30        40        50        60        70        80
|........|.........|.........|.........|.........|.........|.........|.........|
DTTSSKTTEGRKELQKIVDKFESLLTNQTLWRTEGRQEISSTATTILRDVESKVLETALKDPEQKVLKIQNDSVAIETQA
________________________________________________________________________________
cccccccccchhhhhhhhhhhhhhhhhcccceeecccccceexcccxxehhhhhhhhhhhhhhhcccccheeeeccccceeeeeeex
22111121223445555555544322222243433322223454444444444344444333443333433122122221

81       90        100       110       120       130       140       150       160
|........|.........|.........|.........|.........|.........|.........|.........|
ITDNCSEERKTFNLNVQMNSMDIRCSDIIQGDTQGPSAIAFISYSSLGNIINATFFEEMDKKDQVYLNSQVVSAAIGPKR
________________________________________________________________________________
xcccccccccceeeeeeeeeccccccceeeeeeeccccccccceeeeeeeecccchhhhhhhhhhhccccxeeeeecxeeeeeeccccc
12223223222233211112222232223333222122222111223333221123333322221222122221322322

161      170       180       190       200       210       220       230       240
|........|.........|.........|.........|.........|.........|.........|.........|
NVSLSKSVTLTFQHVKMTPSTKKVFCVYWKSTGQGSQWSRDGCFLIHVNKSHTMCNCSHLSSFAVLMALTSQ
________________________________________________________________________
ceeecccceeeeeeeeeeeccccccceeeeeeeeeccccccccccccceeeeeeecccccccccccccchhheeeeehccc
2333212333322222122333323333332232333333443232222212221221211221111121221
```

# mEMR4

VNKSKNTGSKTHTLGVLSEFKSKEEVAKGATKLLRKVEHHILNENSDIPKKDENPLLDIVYET
KRCKTMTLLEAGNNTMKVDCTSGFKEHNSGGETAVAFIAYKSLGNLLNGSFFSNEEGFQEVT
LNSHIVSGAIRSEVKPVLSEPVLLTLQNIQPIDSRAEHLCVHWEGSEEGGSWSTKGCSHVYTNN
SYTICKCFHLSSFAVLMALPHEEDGV

```
1        10        20        30        40        50        60        70        80
|.........|.........|.........|.........|.........|.........|.........|.........|
VNKSKNTGSKTHTLGVLSEFKSKEEVAKGATKLLRKVEHHILNENSDIPKKDENPLLDIVYETKRCKTMTLLEAGNNTMK
________________________AAAAAAAAAAAA____________________________________________
ccccccccccxeeeeeeeexccchhhhhhhhhhhhhhheeeecccccccccccceeeeeecccccceeeeeeccccccee
22334332222111222212223233345555555555433233443222333343333443332211122223443322

81       90        100       110       120       130       140       150       160
|.........|.........|.........|.........|.........|.........|.........|.........|
VDCTSGFKEHNSGGETAVAFIAYKSLGNLLNGSFFSNEEGFQEVTLNSHIVSGAIRSEVKPVLSEPVLLTLQNIQPIDSR
________________________________________________________________________________
eecccccccccccccchheeehhhhhhhhhhccceeccccccxeeeeeeceeeecxeeeexccccccccccceeeeeecccccccccc
21221223333332232221222334444221111112222222122122333344434433222123332322343333

161  170       180       190       200       210       220       230       240
|.........|.........|.........|.........|.........|.........|.........|.........|
AEHLCVHWEGSEEGGSWSTKGCSHVYTNNSYTICKCFHLSSFAVLMALPHEEDGV
________________________________________________________
cceeeeeeeecccccccceecccccceeeeeecccceeeeeeccccchheeeeeeccccccc
22222211212222233323333221122233323322221111111222222222
```

## mEMR1 (F4/80)

RILFKCKEDLILQSEQIQQCQAVQGRDLGYASFCTLVNATFTILDNTCENKSAPVSLQSAATSVSLVLE
QATTWFELSKEETSTLGTILLETVESTMLAALLIPSGNASQMIQTEYLDIESKVINEECKENESINLAA
RGDKMNVGCFIIKESVSTGAPGVAFVSFAHMESVLNERFFEDGQSFRKLRMNSRVVGGTVTGEKKEDFS
KPIIYTLQHIQPKQKSERPICVSWNTDVEDGRWTPSGCEIVEASETHTVCSCNRMANLAIIMASGE

```
1        10        20        30        40        50        60        70        80
|........|.........|.........|.........|.........|.........|.........|.........|
DLILQSEQIQQCQAVQGRDLGYASFCTLVNATFTILDNTCENKSAPVSLQSAATSVSLVLEQATTWFELSKEETSTLGTI
________________________________________________________________________________
ccccchhhhhhhhhhcccccccxxeeeexxxeeeeeecccccccccceexxcccccehhhhhhcccxeexcccccccccee
3233232111112222112122322322333343333221111123322222333333333343333222332232223

81       90        100       110       120       130       140       150       160
|........|.........|.........|.........|.........|.........|.........|.........|
LLETVESTMLAALLIPSGNASQMIQTEYLDIESKVINEECKENESINLAARGDKMNVGCFIIKESVSTGAPGVAFVSFAH
________________________________________________________________________________
ehhhhhhhhhhheeccccccchhhhhhhxxhhhhhhhhhhcccccxxxhhhccccccceeeeeeeeeeccccccccceeeeeehh
33332222111111211112323223322244433344433211244444332232122332332333312111111111

161      170       180       190       200       210       220       230       240
|........|.........|.........|.........|.........|.........|.........|.........|
MESVLNERFFEDGQSFRKLRMNSRVVGGTVTGEKKEDFSKPIIYTLQHIQPKQKSERPICVSWNTDVEDGRWTPSGCEIV
________________________________________________________________________________
hhhhhhhhhccccchhhhheecceeeeeeeeeecccccccccceeeeeeecccccccccccceeeeeeccccccccccccccceee
23222222334544433333555544423333221112233234444433222232443344333222222211233453233

241      250       260       270       280       290       300       310       320
|........|.........|.........|.........|.........|.........|.........|.........|
EASETHTVCSCNRMANLAIIMASGE
_________________________
excccceeeexhhhhhhhhhhhhccc
3221111334434433331111123
```

GPR56

GPS (343-389)

MTPQSLLQTTLFLLSLLFLVQGAHGRGHREDFRFCSQRNQTHRSSLHYKPTPDLRISIENSEEALTVHAPFPAAHP
ASRSFPDPRGLYHFCLYWNRHAGRLHLLYGKRDFLLSDKASSLLCFQHQEESLAQGPPLLATSVTSWWSPQNISLP
SAASFTFSFHSPPHTAAHNASVDMCELKRDLQLLSQFLKHPQKASRRPSAAPASQQLQSLESKLTSVRMGDMVSFE
EDRINATVWKLQPTAGLQDLHIHSRQEEEQSEIMEYSVLLPRTLFQRTKGRSGEAEKRLLLVDFSSQALFQDKNSS
QVLGEKVLGIVVQNTKVANLTEPVVLTFQHQLQPKNVTLQCVFWVEDPTLSSPGHWSSAGCETVRRETQTSCFCNH
LTYFAVLMVSSVEV

```
1       10        20        30        40        50        60        70        80
|........|.........|.........|.........|.........|.........|.........|.........|
MTPQSLLQTTLFLLSLLFLVQGAHGRGHREDFRFCSQRNQTHRSSLHYKPTPDLRISIENSEEALTVHAPFPAAHPASRS
________________________________________________________________________________
ccchhhhhhhhhhhhhheeecccccccccchxeeeeccccccccccceeeccccccceeeeeecccchheeecccccccccccc
11122222322222112222222214423555543332232111222222223222322233322221221112223344544

81      90        100       110       120       130       140       150       160
|........|.........|.........|.........|.........|.........|.........|.........|
FPDPRGLYHFCLYWNRHAGRLHLLYGKRDFLLSDKASSLLCFQHQEESLAQGPPLLATSVTSWWSPQNISLPSAASFTFS
________________________________________________________________________________
cccccceeeeeeeeeccccccceeeeeeccccceeeccccchxeeehcchhhhcccccceeeeeeecxcccccccccccccceeeeee
3333333344443344444443433222133331233222211223322233222222333221111111111222222

161     170       180       190       200       210       220       230       240
|........|.........|.........|.........|.........|.........|.........|.........|
FHSPPHTAAHNASVDMCELKRDLQLLSQFLKHPQKASRRPSAAPASQQLQSLESKLTSVRMGDMVSFEEDRINATVWKLQ
________________________________________________________________________________
eccccccccccccchhhhhhhhhhhhhhhhhhcccccccccccchhhhhhhhhhccxeeeccccceechhhhhhheeexc
22223322212223133333445555455554555544433222233333343432222233323233222244432222

241     250       260       270       280       290       300       310       320
|........|.........|.........|.........|.........|.........|.........|.........|
PTAGLQDLHIHSRQEEEQSEIMEYSVLLPRTLFQRTKGRSGEAEKRLLLVDFSSQALFQDKNSSQVLGEKVLGIVVQNTK
________________________________________________________________________________
ccccccccceecccchhhhhhhhhhheexcchhhhhccccccchhhhheeeeeecccchhhhccccccchhxxxeeeeeeeeccc
21122113322222222332221224333333343233423334432222222223221212112223323324434222222

321     330       340       350       360       370       380       390       400
|........|.........|.........|.........|.........|.........|.........|.........|
```

VANLTEPVVLTFQHQLQPKNVTLQCVFWVEDPTLSSPGHWSSAGCETVRRETQTSCFCNHLTYFAVLMVSSVEV

______________________________________________________________________________

ccccccceeeeeecccccccceeeeeeeeecccccccccccccccccxxxcccccchhhhxxeeeeeeexxxxc

22343333323332111111221222222211111122222222224544444433212223333222222211222

GPR97 GAIN + GPS (211-256)

QEKPTEGPRNTCLGSNNMYDIFNLNDKALCFTKCRQSGSDSCNVENLQRYWLNYEAHLMKE
GLTQKVNTPFLKALVQNLSTNTAEDFYFSLEPSQVPRQVMKDEDKPPDRVRLPKSLFRSLPGNR
SVVRLAVTILDIGPGTLFKGPRLGLGDGSGVLNNRLVGLSVGQMHVTKLAEPLEIVFSHQRPPP
NMTLTCVFWDVTKGTTGDWSSEGCSTEVRPEGTVCCCDHLTFFALLLRPTL

```
1        10        20        30        40        50        60        70        80
|........|.........|.........|.........|.........|.........|.........|.........|
QEKPTEGPRNTCLGSNNMYDIFNLNDKALCFTKCRQSGSDSCNVENLQRYWLNYEAHLMKEGLTQKVNTPFLKALVQNLS
________________________________________________________________________________
ccccccccccceeecccccceehhcccccceeeeeecccccccccchhhhhhhhhhhhhhhhhhccccccccchhhhhhhhhhc
11234332222222122332233333233011123322322113343444333333222222223233433324443223

81       90        100       110       120       130       140       150       160
|........|.........|.........|.........|.........|.........|.........|.........|
TNTAEDFYFSLEPSQVPRQVMKDEDKPPDRVRLPKSLFRSLPGNRSVVRLAVTILDIGPGTLFKGPRLGLGDGSGVLNNR
________________________________________________________________________________
ccccccceeeecccccccchhexccccccccccceecchhhhhcccccccceeeeeeeeeeccccccceecccccceeccccccceecce
33312332210133335444444444422222344554432224444455544422222223333333322212222111

161   170        180       190       200       210       220       230       240
|........|.........|.........|.........|.........|.........|.........|.........|
LVGLSVGQMHVTKLAEPLEIVFSHQRPPPNMTLTCVFWDVTKGTTGDWSSEGCSTEVRPEGTVCCCDHLTFFALLLRPTL
________________________________________________________________________________
eeeeecccceeehhhcccxeeeeeecccccccccceeeeeeeeecccccccccccccccccceeecccccceeeeecccchhhhhhhhcccc
11223112222333333332333322222221112233333222211122112222222221122111112333333234
```

GPR114

MDHCGALFLCLCLLTLQNATTETWEELLSYMENMQVSRGRSSVFSSRQLHQLEQMLLNTSFPGYNLTLQ

TPTIQSLAFKLSCDFSGLSLTSATLKRVPQAGGQHARGQHAMQFPAELTRDACKTRPRELRLICIYFSN

THFFKDENNSSLLNNYVLGAQLSHGHVNNLRDPVNISFWHNQSLEGYTLTCVFWKEGARKQPWGGWSPE

GCRTEQPSHSQVLCRCNHLTYFAVLMQLSPALVPAEL

```
1         10        20        30        40        50        60        70        80
|........|.........|.........|.........|.........|.........|.........|.........|
MDHCGALFLCLCLLTLQNATTETWEELLSYMENMQVSRGRSSVFSSRQLHQLEQMLLNTSFPGYNLTLQTPTIQSLAFKL
________________________________________________________________________________
cccchhhhhhhxxxxhcccchhhhhhhhhhhhhhhccccccceeechhhhhhhhhhhhhccccccceecccccccchhheeee
22332221111122122223333334444322221112333124423344554432212221111222112212333333

81        90        100       110       120       130       140       150       160
|........|.........|.........|.........|.........|.........|.........|.........|
SCDFSGLSLTSATLKRVPQAGGQHARGQHAMQFPAELTRDACKTRPRELRLICIYFSNTHFFKDENNSSLLNNYVLGAQL
________________________________________________________________________________
cccccccceechhhhhhhhhcccccccchhhhhxcchhhhhhhhhcccccxeeeeeeeeeccccceeccccccccchhhhheeheec
33221111123434444555543322212233344445533434434422443431123113232211232123221111

161       170       180       190       200       210       220       230       240
|........|.........|.........|.........|.........|.........|.........|.........|
SHGHVNNLRDPVNISFWHNQSLEGYTLTCVFWKEGARKQPWGGWSPEGCRTEQPSHSQVLCRCNHLTYFAVLMQLSPALV
________________________________________________________________________________
cccccccccccceeeeeccccccccceeeeeeecccccccccccccccccccccccccceeexcchhhhhhhhhcccccc
22333344444332111221121221133324433333222122332332232331333333322212222222233211

241       250       260       270       280       290       300       310       320
|........|.........|.........|.........|.........|.........|.........|.........|
PAEL
____
cccc
1112
```

Rat CL1 (latrophilin-1)

APSTRRPPAPNLHVSPELFCEPREVRRVQWPATQQGMLVERPCPKGTRGIASFQCLPALGLWN
PRGPDLSNCTSPWVNQVAQKIKSGENAANIASELARHTRGSIYAGDVSSSVKLMEQLLDILDA
QLQALRPIERESAGKNYNKMHKRERTCKDYIKAVVETVDNLLRPEALESWKDMNATEQVHT
ATMLLDVLEEGAFLLADNVREPARFLAAKQNVVLEVTVLSTEGQVQELVFPQEYASESSIQLS
ANTIKQNSRNGVVKVVFILYNNLGLFLSTENATVKLAGEAGTGGPGGASLVVNSQVIAASINK
ESSRVFLMDPVIFTVAHLEAKNHFNANCSFWNYSERSMLGYWSTQGCRLVESNKTHTTCACS
HLTNFAVLMAHREIY

```
1        10        20        30        40        50        60        70        80
|........|.........|.........|.........|.........|.........|.........|.........|
APSTRRPPAPNLHVSPELFCEPREVRRVQWPATQQGMLVERPCPKGTRGIASFQCLPALGLWNPRGPDLSNCTSPWVNQV
________________________________________________________________________________
cccccccccccccccccccccccchheeeccccccceeecccccccccceeecxccccccccccccccccccccchhhhh
23322222222112222233344444443332111333233355554443333111111221221222222233333344

81       90        100       110       120       130       140       150       160
|........|.........|.........|.........|.........|.........|.........|.........|
AQKIKSGENAANIASELARHTRGSIYAGDVSSSVKLMEQLLDILDAQLQALRPIERESAGKNYNKMHKRERTCKDYIKAV
________________________________________________________________________________
hhhhhccchhhhhhhhhhhhcccceeecccchhhhhhhhhhhhhhhhhhhhhhhhhhhcccchhhhcccchhhhhhhhhh
44333332333334445444333322212222233344454333334444445543311223444333223333554444

161      170       180       190       200       210       220       230       240
|........|.........|.........|.........|.........|.........|.........|.........|
VETVDNLLRPEALESWKDMNATEQVHTATMLLDVLEEGAFLLADNVREPARFLAAKQNVVLEVTVLSTEGQVQELVFPQE
________________________________________________________________________________
hhhhhhhccchhhhhhhhcchhhhhhhhhhhhhhhhhhhhhhhhhhchhhhhhhhhccceeeeeeeeccccccxeeecccc
44455443322222332222222222222333333332222333355554332222111121111112222211112111

241      250       260       270       280       290       300       310       320
|........|.........|.........|.........|.........|.........|.........|.........|
YASESSIQLSANTIKQNSRNGVVKVVFILYNNLGLFLSTENATVKLAGEAGTGGPGGASLVVNSQVIAASINKESSRVFL
________________________________________________________________________________
ccccccxeexcccchhccccceeeeeeeexccccceeccccceeeeexccccccccceeeeeeeeeeeexcccccceeee
11122122233332344433232223333312122211112222223233322222222211111112222444432321

321      330       340       350       360       370       380       390       400
```

|........|.........|.........|.........|.........|.........|.........|.........|

MDPVIFTVAHLEAKNHFNANCSFWNYSERSMLGYWSTQGCRLVESNKTHTTCACSHLTNFAVLMAHREIY

______________________________________________________________________

eccceeeexcccxcccccccccccchhhhhhxceeecccccceeeecccccceexhhhhhhhhhhhhhhccc

2211222122112211222222223333322222222333443333222111122223222222222233345

## Rat Latrophilin-2

ENAASLANELAKHTKGTVFAGDVSSSVRLMEQLVDILDAQLQELKPSEKDSAGRSYNKLQKREKTCRAY
LKAIVDTVDNLLRAETLDCWKHMNSSEQAHTATMLLDTLEEGAFVLADNLLEPTRVSMPTDNIVLEVAV
LSTEGQVQDFTFHLGFKGAFSSIQLSANTVKQNSRNGLAKVVFIIYRSLGPFLSTENATVKLGADLLGR
NSTIAVNSHVLSVSINKESSRVYLTDPVLFSMPHIDSDNYFNANCSFWNYSERTMMGYWSTQGCKLVDT
NKTRTTCACSHLTNFAILMAHREI

```
1        10        20        30        40        50        60        70        80
|........|.........|.........|.........|.........|.........|.........|.........|
ENAASLANELAKHTKGTVFAGDVSSSVRLMEQLVDILDAQLQELKPSEKDSAGRSYNKLQKREKTCRAYLKAIVDTVDNL
________________________________________________________________________________
cchhhhhhhhhhcccceeeecccchhhhhhhhhhhhhhhhhhhhhccccccccchhhhhhhhhhhhhhhhhhhhhhhhhhh
44434344344332212222222333344454554333333322223221122344554433212223544444455544

81       90        100       110       120       130       140       150       160
|........|.........|.........|.........|.........|.........|.........|.........|
LRAETLDCWKHMNSSEQAHTATMLLDTLEEGAFVLADNLLEPTRVSMPTDNIVLEVAVLSTEGQVQDFTFHLGFKGAFSS
________________________________________________________________________________
hhhhhhhhhhhhccccchhhhhhhhhhhhhhhhhhhhhhcccccccceeccccceeeeeeeeccccccceeeeeeecccccce
33212212322322212212223333333322222332343322123332222222221112211122221223233332

161      170       180       190       200       210       220       230       240
|........|.........|.........|.........|.........|.........|.........|.........|
IQLSANTVKQNSRNGLAKVVFIIYRSLGPFLSTENATVKLGADLLGRNSTIAVNSHVLSVSINKESSRVYLTDPVLFSMP
______________________AAAA______________________________________________________
eeecccccchccccccceeeeeeeeccccccccccccheeeechhhccccceeeeecceeeeeeeccccceeeecccceeeecc
22333323444333322334444333443311122222223344333333322111111222133333222122112221 2

241      250       260       270       280       290       300       310       320
|........|.........|.........|.........|.........|.........|.........|.........|
HIDSDNYFNANCSFWNYSERTMMGYWSTQGCKLVDTNKTRTTCACSHLTNFAILMAHREI
____________________________________________________________
ccccccccccccccccccccxeeeeeccccceeeeeccccceeexchhhhhhhhhhhccc
111111112222222333333222221223333222221111112223222222223344 35
```

hELTD1

MCVPGFRSSSNQDRFITNDGTVCIENVNANCHLDNVCIAANINKTLTKIRSIKEPVALLQEVYRNSVTD
LSPTDIITYIEILAESSSLLGYKNNTISAKDTLSNSTLTEFVKTVNNFVQRDTFVVWDKLSVNHRRTHL
TKLMHTVEQATLRISQSFQKTTEFDTNSTDIALKVFFFDSYNMKHIHPHMNMDGDYINIFPKRKAAYDS
NGNVAVAFLYYKSIGPLLSSSDNFLLKPQNYDNSEEEERVISSVISVSMSSNPPTLYELEKITFTLSHR
KVTDRYRSLCAFWNYSPDTMNGSWSSEGCELTYSNETHTSCRCNHLTHFAILMSSGPSIGIKDY

```
1       10        20        30        40        50        60        70        80
|........|.........|.........|.........|.........|.........|.........|.........|
MCVPGFRSSSNQDRFITNDGTVCIENVNANCHLDNVCIAANINKTLTKIRSIKEPVALLQEVYRNSVTDLSPTDIITYIE
________________________________________________________________________________
cccccccccccccceeeccccceeeeeeccccccccccceeeeexccccccehhhcccchhhhhhhhcccccccccccxeehhh
22122332444433333332333222221122222221223345445333443553434444444433221112223333

81      90        100       110       120       130       140       150       160
|........|.........|.........|.........|.........|.........|.........|.........|
ILAESSSLLGYKNNTISAKDTLSNSTLTEFVKTVNNFVQRDTFVVWDKLSVNHRRTHLTKLMHTVEQATLRISQSFQKTT
________________________________________________________________________________
hhhhhccxeccccccceeeeccccccchhhhhhhhhhhhhcccceeeeeeecccccccchhhhhhhhhhhhhhhhhehhhhccce
22233221112212222222222212233445555554333333342213443211211134444334344444454332

161     170       180       190       200       210       220       230       240
|........|.........|.........|.........|.........|.........|.........|.........|
EFDTNSTDIALKVFFFDSYNMKHIHPHMNMDGDYINIFPKRKAAYDSNGNVAVAFLYYKSIGPLLSSSDNFLLKPQNYDN
________________________________________________________________________________
eeccccchhheeeeeeccccccccccccccccccccceeeeeccccceccccccccceeeeeeeeeccccceecccccccccceccccccccc
22223222332221221112212332222222333322121122133112211112222233333323322222221122
241     250       260       270       280       290       300       310       320
|........|.........|.........|.........|.........|.........|.........|.........|
SEEEERVISSVISVSMSSNPPTLYELEKITFTLSHRKVTDRYRSLCAFWNYSPDTMNGSWSSEGCELTYSNETHTSCRCN
________________________________________________________________________________
chhhhheeeeeeeeeeeccccccchhhhhheeeeeeccccccchhhhhhheexcccccccccccccccccceeeeeccccccccceccc
22333444444321111112212232233331223333455333324422233222211112222111111133221221
321     330       340       350       360       370       380       390       400
|........|.........|.........|.........|.........|.........|.........|.........|
HLTHFAILMSSGPSIGIKDY
____________________
hhheeeeeeecccccccceecc
22122222222132222233
```

## BAI-1

AKAQRGLPGEGVSEVIQTLVEISQDGTSYSGDLLSTIDVLRNMTEIFRRAYYSPTPGDVQNFVQILSNL
LAEENRDKWEEAQLAGPNAKELFRLVEDFVDVIGFRMKDLRDAYQVTDNLVLSIHKLPASGATDISFPM
KGWRATGDWAKVPEDRVTVSKSVFSTGLTEADEASVFVVGTVLYRNLGSFLALQRNTTVLNSKVISVTV
KPPPRSLRTPLEIEFAHMYNGTTNQTCILWDETDVPSSSAPPQLGPWSWRGCRTVPLDALRTRCLCDRL
STFAILAQLSADAN

```
1        10        20        30        40        50        60        70        80
|........|.........|.........|.........|.........|.........|.........|.........|
AKAQRGLPGEGVSEVIQTLVEISQDGTSYSGDLLSTIDVLRNMTEIFRRAYYSPTPGDVQNFVQILSNLLAEENRDKWEE
________________________________________________________________________________
cccccccccchhhhhhhhhhhhcccccccccccxxhhhhhhhhhhhhhhhccccccchhhhhhhhhhhhhhhcchhhhhh
23332322122334444344333322222222222233344455554444332212223233444332233222222122

81       90        100       110       120       130       140       150       160
|........|.........|.........|.........|.........|.........|.........|.........|
AQLAGPNAKELFRLVEDFVDVIGFRMKDLRDAYQVTDNLVLSIHKLPASGATDISFPMKGWRATGDWAKVPEDRVTVSKS
_____________AAAAAA_____________________________________________________________
hhhcccchhhhhhhhhhhhhhheehhhhhhhhhhhhheeeeeecccccccccceecccccccccccccccccceeeeeeee
21222334444555555532222222221224434422333333222333311222233333334434323321211112

161     170       180       190       200       210       220       230       240
|........|.........|.........|.........|.........|.........|.........|.........|
VFSTGLTEADEASVFVVGTVLYRNLGSFLALQRNTTVLNSKVISVTVKPPPRSLRTPLEIEFAHMYNGTTNQTCILWDET
________________________________________________________________________________
eecccccccccceeeeeeeeeeeecccchhhhhxccceeeecceeeeeeeecccccccccccceeeeeeexxcccccceeeeeeccc
22222332212111111332233333232222223222211122333333444444432211221122111111111112

241     250       260       270       280       290       300       310       320
|........|.........|.........|.........|.........|.........|.........|.........|
DVPSSSAPPQLGPWSWRGCRTVPLDALRTRCLCDRLSTFAILAQLSADAN
__________________________________________________
ccccccccccccccccccccccccccccxxhhhhhhhhhhhhhhhhhccc
21111112222133343333333332111123233422221112222222
```

## hBAI2

LYLSLREHLAKGQRMLAGEGMSQVVRSLQELLARRTYYSGDLLFSVDILRNVTDTFKRATYVPSADDVQ

RFFQVVSFMVDAENKEKWDDAQQVSPGSVHLLRVVEDFIHLVGDALKAFQSSLIVTDNLVISIQREPVS

AVSSDITFPMRGRRGMKDWVRHSEDRLFLPKEVLSLSSPGKPATSGAAGSPGRGRGPGTVPPGPGHSHQ

RLLPADPDESSYFVIGAVLYRTLGLILPPPRPPLAVTSRVMTVTVRPPTQPPAEPLITVELSYIINGTT

DPHCASWDYSRADASSGDWDTENCQTLETQAAHTRCQCQHLSTFAVLAQPPKDLTLELAG

```
1       10        20        30        40        50        60        70        80
|........|.........|.........|.........|.........|.........|.........|.........|
LYLSLREHLAKGQRMLAGEGMSQVVRSLQELLARRTYYSGDLLFSVDILRNVTDTFKRATYVPSADDVQRFFQVVSFMVD
___A____________________________________________________________________________
ccchhhhhhhhhhhhhhhcchhhhhhhhhhhhhhhhcccccceeeeeehhhhhhhhhxcceeccccchhhhhhheeeeeex
12222222444433322122444445545432223333322211223344555554433322123344454344432332

81      90        100       110       120       130       140       150       160
|........|.........|.........|.........|.........|.........|.........|.........|
AENKEKWDDAQQVSPGSVHLLRVVEDFIHLVGDALKAFQSSLIVTDNLVISIQREPVSAVSSDITFPMRGRRGMKDWVRH
_____________________AAAAAAAAAAA____________________________________________A___
ccccccccccccccccccceeeehhhhhhhhhhhhhhhhhccxeeeecceeeeecccccxecccceeeccccccchhhhhc
21111122322222223333444555544444444333222222112233333333323322233122344445555434

161     170       180       190       200       210       220       230       240
|........|.........|.........|.........|.........|.........|.........|.........|
SEDRLFLPKEVLSLSSPGKPATSGAAGSPGRGRGPGTVPPGPGHSHQRLLPADPDESSYFVIGAVLYRTLGLILPPPRPP
________________________________________________________________________________
ccccccccccceeeecccccccccccccccccccccccccccccccccccceeccccccccceeeeeeehhhxcceecccccc
32211111122333222333222212211111111132111222333222222111111221222333333332222222
241     250       260       270       280       290       300       310       320
|........|.........|.........|.........|.........|.........|.........|.........|
LAVTSRVMTVTVRPPTQPPAEPLITVELSYIINGTTDPHCASWDYSRADASSGDWDTENCQTLETQAAHTRCQCQHLSTF
________________________________________________________________________________
ceeeeeeeeeeeecccccccccceeeeeeeeeeeccccccccccccccccccccccccchhhhhhhhhcxxhhhcccxe
33322222444433223332222122222222233222111112222222222221111122212222211111222332122
321     330       340       350       360       370       380       390       400
|........|.........|.........|.........|.........|.........|.........|.........|
AVLAQPPKDLTLELAG
________________
eeecccccceeeeeccc
2223332332222222
```

hBAI3

HSIKEHLAKGQRMLAGDGMSQVTKTLLDLTQRKNFYAGDLLMSVEILRNVTDTFKRASYIPA
SDGVQNFFQIVSNLLDEENKEKWEDAQQIYPGSIELMQVIEDFIHIVGMGMMDFQNSYLMTGN
VVASIQKLPAASVLTDINFPMKGRKGMVDWARNSEDRVVIPKSIFTPVSSKELDESSVFVLGA
VLYKNLDLILPTLRNYTVINSKIIVVTIRPEPKTTDSFLEIELAHLANGTLNPYCVLWDDSKTNE
SLGTWSTQGCKTVLTDASHTKCLCDRLSTFAILAQQPREII

```
1        10        20        30        40        50        60        70        80
|........|.........|.........|.........|.........|.........|.........|.........|
HSIKEHLAKGQRMLAGDGMSQVTKTLLDLTQRKNFYAGDLLMSVEILRNVTDTFKRASYIPASDGVQNFFQIVSNLLDEE
___AA___________________________________________________________________________
cchhhhhhhhhhhhcccchhhhhhhhhhhhhhhhhcchhhhhhhhhhhhhhhhhcccccccccchhhhhhhhhhhhhhhh
45444344443332212234444434433222112221222222334455555443332221233444444444333322

81       90        100       110       120       130       140       150       160
|........|.........|.........|.........|.........|.........|.........|.........|
NKEKWEDAQQIYPGSIELMQVIEDFIHIVGMGMMDFQNSYLMTGNVVASIQKLPAASVLTDINFPMKGRKGMVDWARNSE
________________________________________________________________________________
hhhhhhhhhhcccccchhhhhhhhhhhheeeeeecccccceeeccchhhhhhhcccccceeeccccccccccccceeehhcccc
2222223222222223222334444433322222222222122211213333322111222223312232223444433432

161      170       180       190       200       210       220       230       240
|........|.........|.........|.........|.........|.........|.........|.........|
DRVVIPKSIFTPVSSKELDESSVFVLGAVLYKNLDLILPTLRNYTVINSKIIVVTIRPEPKTTDSFLEIELAHLANGTLN
________________________________________________________________________________
cceeecccceecccccccccccxeeeehhhhhhhhhhhhcccccceeeecccceeeeeeecccccccchhhhhhhhhhhccccc
21111112332222222211221111122222333334343232222331111333333223432333211111212332222

241      250       260       270       280       290       300       310       320
|........|.........|.........|.........|.........|.........|.........|.........|
PYCVLWDDSKTNESLGTWSTQGCKTVLTDASHTKCLCDRLSTFAILAQQPREII
______________________________________________________
ceeeeecccccccccccccccccccceeeeecccccchhhhhhhhhhhhhhcchhhcc
222222111212222222233333233332111223333332222222333444454
```

CELSR1 GAIN + GPS (2407-2460)

DLRAMNEKLSRNETQVDGARALQLVRALRSATQHTGTLFGNDVRTAYQLLGHVLQHESWQQGFDLAATQDADFHEDVIHSGSALLA

PATRAAWEQIQRSEGGTAQLLRRLEGYFSNVARNVRRTYLRPFVIVTANMILAVDIFDKFNFTGARVPRFDTIHEEFPRELESSVS

FPADFFRPPEEKEGPLLRPAGRRTTPQTTRPGPGTEREAPISRRRRHPDDAGQFAVALVIIYRTLGQLLPERYDPDRRSLRLPHRP

IINTPMVSTLVYSEGAPLPRPLERPVLVEFALLEVEERTKPVCVFWNHSLAVGGTGGWSARGCELLSRNRTHVACQCSHTASFAVL

MDISRR

```
1       10        20        30        40        50        60        70        80
|........|.........|.........|.........|.........|.........|.........|.........|
DLRAMNEKLSRNETQVDGARALQLVRALRSATQHTGTLFGNDVRTAYQLLGHVLQHESWQQGFDLAATQDADFHEDVIHS
________________________________________________________________________________
cchhhhhhccccchhhhhhhhhhhhhhhhhccccccecccchhhhhhhhhhhhcxxhhhhhhhhhhcccccchheehc
44344443233332113322333334454443332222333333333443322121112233322112112222233223

81      90        100       110       120       130       140       150       160
|........|.........|.........|.........|.........|.........|.........|.........|
GSALLAPATRAAWEQIQRSEGGTAQLLRRLEGYFSNVARNVRRTYLRPFVIVTANMILAVDIFDKFNFTGARVPRFDTIH
________________________________________________________________________________
cccccchhhhhhhhhhhhhcchhhhhhhhhhhhhhhhhhhhhhhcccccceeeexxxeeeexxxxcccccccccccccccc
22212332333344433222223333334444444445554544333322221111111232233233343222222334

161     170       180       190       200       210       220       230       240
|........|.........|.........|.........|.........|.........|.........|.........|
EEFPRELESSVSFPADFFRPPEEKEGPLLRPAGRRTTPQTTRPGPGTEREAPISRRRRHPDDAGQFAVALVIIYRTLGQL
________________________________________________________________________________
hhcchhhxxxcccccccccccccccccccccccccccccccccccccccccccccccccccchhhhheehhhhhhhhh
44443443332223333332222222223333333244432223333112321112232224322221122223333422

241     250       260       270       280       290       300       310       320
|........|.........|.........|.........|.........|.........|.........|.........|
LPERYDPDRRSLRLPHRPIINTPMVSTLVYSEGAPLPRPLERPVLVEFALLEVEERTKPVCVFWNHSLAVGGTGGWSARG
________________________________________________________________________________
ccccccccccxxccccccccccccceeeeeecccccccccccccccxeehhhhhhhhcccccceeeeeeecceecccccccccccc
22112223244445444322221111111111333344444332222112211222211112211111111112212222

321     330       340       350       360       370       380       390       400
|........|.........|.........|.........|.........|.........|.........|.........|
CELLSRNRTHVACQCSHTASFAVLMDISRR
______________________________
```

xxhhccccceeeeccccchhheeeeecccc

442322222111122111111113343455

mGPR124 (ADGRA2)

NDITRVLYTFVLMPINASNALTLAHQLRVYTAEAASFSDMMDVVYVAQMIQKFLGYVDQIKELVEVMVD
MASNLMLVDEHLLWLAQREDKACSGIVGALERIGGAALSPHAQHISVNSRNVALEAYLIKPHSYVGLTC
TAFQRREVGVSGAQPSSVGQDAPVEPEPLADQQLRFRCTTGRPNISLSSFHIKNSVALASIQLPPSLFS
TLPAALAPPVPPDCTLQLLVFRNGRLFRSHGNNTSRPGAAGPGKRRGVATPVIFAGTSGCGVGNLTEPV
AVSLRHWAEGADPMAAWWNQDGPGGWSSEGCRLRYSQPNVSSLYCQHLGNVAVLMELNAFPREAGGSGA
GLHPV

```
1        10        20        30        40        50        60        70        80
|........|.........|.........|.........|.........|.........|.........|.........|
NDITRVLYTFVLMPINASNALTLAHQLRVYTAEAASFSDMMDVVYVAQMIQKFLGYVDQIKELVEVMVDMASNLMLVDEH
________________________________________________________________________________
ccxxxeeeeeeeeeecccccchhhhhhhhhheehhhhhhhhhhhhhhhhhhhhhhhhhhhhhhhhhhhhhhhhhhhhhhh
55555544332222221222333222222222331222233332232444444545444444544544443232222222122

81       90        100       110       120       130       140       150       160
|........|.........|.........|.........|.........|.........|.........|.........|
LLWLAQREDKACSGIVGALERIGGAALSPHAQHISVNSRNVALEAYLIKPHSYVGLTCTAFQRREVGVSGAQPSSVGQDA
________________________________________________________________________________
hhhhhhhhhhchchhhhhhhhhhccccccccccceeeeccchhhhhhhxxccccceceeeexchxxxeeeeccccccccccccc
33333333333323344444443444332232221233322222331122222222212233222222212321112222222

161      170       180       190       200       210       220       230       240
|........|.........|.........|.........|.........|.........|.........|.........|
PVEPEPLADQQLRFRCTTGRPNISLSSFHIKNSVALASIQLPPSLFSTLPAALAPPVPPDCTLQLLVFRNGRLFRSHGNN
________________________________________________________________________________
ccccccchhhhheeeecccccceeeeeeccccccxeeeeeecccccccccccccccccccccccccceeeeexcccxeeeecccccc
22222123422211222211222221221112221121122223332233222221221122122124333454454455444

241      250       260       270       280       290       300       310       320
|........|.........|.........|.........|.........|.........|.........|.........|
TSRPGAAGPGKRRGVATPVIFAGTSGCGVGNLTEPVAVSLRHWAEGADPMAAWWNQDGPGGWSSEGCRLRYSQPNVSSLY
________________________________________________________________________________
ccccccccccccccccceeeeeeecccccccccccccccccceeeehhhhccccchhhhhhccccccccccccccceeeeecccccccehh
333322212222222123312221112222233222443334344543333322222122122332221122223312232

321      330       340       350       360       370       380       390       400
|........|.........|.........|.........|.........|.........|.........|.........|
CQHLGNVAVLMELNAFPREAGGSGAGLHPV
```

---

hhhhchhheeehhccccccccccccccccc

223223211111443334333331112223

## hGPR116 (Ig-hepta, ADGRF5)

TFHTGSSSLPAAKEVNKKQVCYKHNFNASSVSWCSKTVDVCCHFTNAANNSVWSPSMKLNLVPGENITC
QDPVIGVGEPGKVIQKLCRFSNVPSSPESPIGGTITYKCVGSQWEEKRNDCISAPINSLLQMAKALIKS
PSQDEMLPTYLKDLSISIDKAEHEISSSPGSLGAIINILDLLSTVPTQVNSEMMTHVLSTVNVILGKPV
LNTWKVLQQQWTNQSSQLLHSVERFSQALQSGDSPPLSFSQTNVQMSSMVIKSSHPETYQQRFVFPYFD
LWGNVVIDKSYLENLQSDSSIVTMAFPTLQAILAQDIQENNFAESLVMTTTVSHNTTMPFRISMTFKNN
SPSGGETKCVFWNFRLANNTGGWDSSGCYVEEGDGDNVTCICDHLTSFSILMSPDSPDPSS

```
1        10         20        30        40        50        60        70        80
|........|.........|.........|.........|.........|.........|.........|.........|
TFHTGSSSLPAAKEVNKKQVCYKHNFNASSVSWCSKTVDVCCHFTNAANNSVWSPSMKLNLVPGENITCQDPVIGVGEPG
________________________________________________________________________________
cccccccccchhhhhccceeeeeecccccceeeeccceeeeeeeecccccccccccccceeeecccccccccceeeeecccc
22233222233333322211111212111122333333333334433332212332111112212111221222332335

81       90         100       110       120       130       140       150       160
|........|.........|.........|.........|.........|.........|.........|.........|
KVIQKLCRFSNVPSSPESPIGGTITYKCVGSQWEEKRNDCISAPINSLLQMAKALIKSPSQDEMLPTYLKDLSISIDKAE
________________________________________________________________________________
chhhhhccccccccccccccceeeeeeecccchhhcccccccchhhhhhhhhhhhhcccccchhhhhhcccxeexccxx
54555554444333332122212222222332121221122134443444455444332221123344333344333334

161      170        180       190       200       210       220       230       240
|........|.........|.........|.........|.........|.........|.........|.........|
HEISSSPGSLGAIINILDLLSTVPTQVNSEMMTHVLSTVNVILGKPVLNTWKVLQQQWTNQSSQLLHSVERFSQALQSGD
________________________________________________________________________________
eeeccccccchhhhhhhhhhhhcccccccccxxeeeeeeeeeeeeeecccccchhhhhhhhhhhcchhhhhhhhhhhhhhhcccc
43322322222333434434444332222322234333333222234444433433323344333555555555444322

241      250        260       270       280       290       300       310       320
|........|.........|.........|.........|.........|.........|.........|.........|
SPPLSFSQTNVQMSSMVIKSSHPETYQQRFVFPYFDLWGNVVIDKSYLENLQSDSSIVTMAFPTLQAILAQDIQENNFAE
________________________________________________________________________________
cccceeecceeeeeeeeeeeccccccccceeeeeeeeexcccceeeeehhhhhhcccccceeeeecchhhhhhhhhhhhhccccx
22222112222222222332222222233222223344333333222212332322221221133333232222322221122222

321      330        340       350       360       370       380       390       400
|........|.........|.........|.........|.........|.........|.........|.........|
```

SLVMTTTVSHNTTMPFRISMTFKNNSPSGGETKCVFWNFRLANNTGGWDSSGCYVEEGDGDNVTCICDHLTSFSILMSPD

________________________________________________________________________________

eeeeeeeecccccccceeeeeeeeecccccccccceeeeeeeeeeeccccccccccccceeeecccccceeeeeeccccccceeeeeeccc

3321111121233222333333222331121112323333233332111223321111112223443432222111111

401 410 420 430 440 450 460 470 480

|........|.........|.........|.........|.........|.........|.........|.........|

SPDPSS

______

cccccc

121221

hVLGR1(GPR98)

FLTNPQGGAQIVEEKDDTGFAAFAMVIITGSDLHNGIIGFSEESQSGLELREGAVMRRLHLIVTRQPNR
AFEDVKVFWRVTLNKTVVVLQKDGVNLVEELQSVSGTTTCTMGQTKCFISIELKPEKVPQVEVYFFVEL
YEATAGAAINNSARFAQIKILESDESQSLVYFSVGSRLAVAHKKATLISLQVARDSGTGLMMSVNFSTQ
ELRSAETIGRTIISPAISGKDFVITEGTLVFEPGQRSTVLDVILTPETGSLNSFPKRFQIVLFDPKGGA
RIDKVYGTANITLVSDADSQAIWGLADQLHQPVNDDILNRVLHTISMKVATENTDEQLSAMMHLIEKIT
TEGKIQAFSVASRTLFYEILCSLINPKRKDTRGFSHFAEVTENFAFSLLTNVTCGSPGEKSKTILDSCP
YLSILALHWYPQQINGHKFEGKEGDYIRIPERLLDVQDAEIMAGKSTCKLVQFTEYSSQQWFISGNNLP
TLKNKVLSLSVKGQSSQLLTNDNEVLYRIYAAEPRIIPQTSLCLLWNQAAASWLSDSQFCKVVEETADY
VECACSHMSVYAVYARTDNLSSY

1 10 20 30 40 50 60 70 80
|.........|.........|.........|.........|.........|.........|.........|.........|
FLTNPQGGAQIVEEKDDTGFAAFAMVIITGSDLHNGIIGFSEESQSGLELREGAVMRRLHLIVTRQPNRAFEDVKVFWRV
________________________________________________________________________________
ccccccccceeeeeeccccchheeeeeeeecccccccceeeeeecccccchhhhhhhhhheeeeecccccccheeeeeee
22222223322222221112122222122122223333322222233112222113333344544433455553445444433

81 90 100 110 120 130 140 150 160
|.........|.........|.........|.........|.........|.........|.........|.........|
TLNKTVVVLQKDGVNLVEELQSVSGTTTCTMGQTKCFISIELKPEKVPQVEVYFFVELYEATAGAAINNSARFAQIKILE
________________________________________________________________________________
ecccceeeeeecccchhhhhhhhccccceeeeeccccceeeeeeeeccccccccceeeeeeeeehhhhhhhhhhcchhhhhheeeec
222223333232344433333221211111111221122222233112232122222222221121244444332221122

161 170 180 190 200 210 220 230 240
|.........|.........|.........|.........|.........|.........|.........|.........|
SDESQSLVYFSVGSRLAVAHKKATLISLQVARDSGTGLMMSVNFSTQELRSAETIGRTIISPAISGKDFVITEGTLVFEP
________________________________________________________________________________
ccccceeeeeeeccccheehhcccceeeeeeeexccccccceeeeeeeccchhhhhhhhcccceeeeeccccccccceeeeeeceeeeecc
22112221122323223332222222332233443222111222334333455554443433222111112222322333

241 250 260 270 280 290 300 310 320
|.........|.........|.........|.........|.........|.........|.........|.........|
GQRSTVLDVILTPETGSLNSFPKRFQIVLFDPKGGARIDKVYGTANITLVSDADSQAIWGLADQLHQPVNDDILNRVLHT
________________________________________________________________________________
cccceeeeeeeccccccccccccccceeeeeeecccccceeeeeeecceeeeeeecccchhhhhhhhhhhcccccchhhhhhhhhe
3322111222222112212344344333212233233443332221122112322222222333334444332334455444

321 330 340 350 360 370 380 390 400

|.........|.........|.........|.........|.........|.........|.........|.........|

ISMKVATENTDEQLSAMMHLIEKITTEGKIQAFSVASRTLFYEILCSLINPKRKDTRGFSHFAEVTENFAFSLLTNVTCG

________________________________________________________________________________

eeeeeccccchhhhhhhhhhhhhhcccccxeeeexxhhhhhhhhhhhccccccccccccxxhhhhhhhhheeeeeeeecc

42312232222222233344444432211111224433323333442332244444454444343333222332322212

401 410 420 430 440 450 460 470 480

|.........|.........|.........|.........|.........|.........|.........|.........|

SPGEKSKTILDSCPYLSILALHWYPQQINGHKFEGKEGDYIRIPERLLDVQDAEIMAGKSTCKLVQFTEYSSQQWFISGN

________________________________________________________________________________

ccccccceeeccccccheeeeexccccccccccccccccceecchhhhhhhhhhhcccccceeeeeecccccceeeeeecc

11222333323332211111111223221111123233222445555323311222122344332223322111122222

481 490 500 510 520 530 540 550 560

|.........|.........|.........|.........|.........|.........|.........|.........|

NLPTLKNKVLSLSVKGQSSQLLTNDNEVLYRIYAAEPRIIPQTSLCLLWNQAAASWLSDSQFCKVVEETADYVECACSHM

________________________________________________________________________________

ccccccxxeeeeeeecccccceeecccchhhhhexcccccccccccheeehhhhhhhhhcccccccxeehhhhhhhhhhhccc

12233332222332111221222333433333444433222331222222222322111123334444433332332221

561 570 580 590 600 610 620 630 640

|.........|.........|.........|.........|.........|.........|.........|.........|

SVYAVYARTDNLSSY

_______________

xeeeeeeeccccccc

112222222222233

| aGPCR | AmphiPath Helix | score |
|---|---|---|
| mEMR1 | - | 0 |
| hEMR2 | + | 12A |
| hEMR3 | - | 0 |
| mEMR4 | + | 12A |
| hCD97 | + | 10A |
| hGPR56 | - | 0 |
| hGPR97 | - | 0 |
| hGPR114 | - | 0 |
| GPR64 | | |
| rLatrophilin-1 | - | 0 |
| rLatrophilin-2 | +/- | 4A |
| | | |
| ELTD1 | - | 0 |
| hBAI1 | + | 6A |
| hBAI2 | + | 12A |
| hBAI3 | +/- | 2A |
| hCelsr1 | - | 0 |
| mGPR124 | - | 0 |
| hGPR116 | - | 0 |
| | | |
| VLGR1 (GPR98) | - | 0 |
